# Supplementary material for: Efficient hybrid numerical modeling of the seismic wavefield in the presence of solid-fluid boundaries
Source: Nat Commun. 2025 Feb 18;16:1722. doi: 10.1038/s41467-025-56530-5 (PMC11836316; doi:10.1038/s41467-025-56530-5)
Supplement: Supplementary file 1 — Supplementary Information [file 41467_2025_56530_MOESM1_ESM.pdf]

Supplementary information for "Efficient hybrid numerical modeling of the seismic wavefield in the presence of solid-fluid boundaries"

Chao Lyu, Barbara Romanowicz, Liang Zhao, Yder Masson

This supplementary file includes seven additional sections, all sharing the same titles as the corresponding main text. The first section presents a detailed mathematical derivation related to the hybrid input and output mirror forces. Subsequently, in the section on 2D hybrid solid-fluid coupling (HSFC) at the Core-Mantle Boundary (CMB), we display global models, numerical wavefields, and waveforms (Figure S1, Figure S2, and Figure S3). Furthermore, the section on 2D HSFC with the Ocean, Crust, and Mantle details global simulations (Figure S4 and Figure S5) and waveform benchmarks for the 2D SORI (Source Outside and Receiver Inside the box) and the section on 3D SORO (Source Outside and Receiver Outside the box) cases (Figure S6, Figure S7, and Figure S8) are listed. Then, in alignment with the discussion section in the main text, we present sections on the waveform convergence of HSFC (Figure S9 and Figure S10) and integrity (FigureS11). Finally, animations related to all numerical simulations in the main text are listed.

1 The stability of solid-fluid coupling in SEM and Derivation of the mathematical expressions of the hybrid input and output mirror forces

In this section, we first summarize how to solve the solid-fluid coupled system, and then illustrate the details of how to obtain the hybrid input and output mirror forces.

The numerical simulation of acoustic and elastic equations, along with the continuous interface conditions of normal displacement and traction at the solid-fluid coupling interface, has been carefully selected and performed to maintain stability. In the discrete system (equation (16)) we constructed for equations (12) and (13), the mass matrices for both the elastic wave equation and the acoustic wave equation are diagonal and inherently symmetric. A second-order central difference method is used in the time direction. The process involves:

- (i) first updating the displacement and displacement potential at time step $n+1$ using the known displacement and displacement potential from time steps $n-1$ and n, as well as the solid and fluid accelerations at time step n;
- (ii) calculating the fluid acceleration at time step $n+1$ using the displacement at the same time step;
- (iii) calculating the solid acceleration at time step $n+1$ using the fluid acceleration at that step.

All operations related to the hybrid simulation involve only adding precomputed mirror forces, so they do not impact the stability of the solid-fluid coupling. To further analyze the stability of the solid-fluid coupling, first, let's define the global unknown vector, global mass matrix, and global stiffness matrix as follows:

$$\boldsymbol{U} = \begin{bmatrix} \ddot{\Phi}_f \\ \ddot{\Phi}_b \\ \ddot{\boldsymbol{U}}_b \\ \ddot{\boldsymbol{U}}_s \end{bmatrix}, \tag{S1}$$

$$\boldsymbol{M}^g = \begin{bmatrix} \boldsymbol{M}_{ff} & \boldsymbol{M}_{fb} & 0 & 0 \\ \boldsymbol{M}_{bf} & \boldsymbol{M}_{bb}^f & 0 & 0 \\ 0 & \boldsymbol{A}^T & \boldsymbol{M}_{bb}^s & \boldsymbol{M}_{bs} \\ 0 & 0 & \boldsymbol{M}_{sb} & \boldsymbol{M}_{ss} \end{bmatrix}, \tag{S2}$$

and

$$\boldsymbol{K}^g = \begin{bmatrix} \boldsymbol{K}_{ff} & \boldsymbol{K}_{fb} & 0 & 0 \\ \boldsymbol{K}_{bf} & \boldsymbol{K}_{bb}^f & -\boldsymbol{A} & 0 \\ 0 & 0 & \boldsymbol{K}_{bb}^s & \boldsymbol{K}_{bs} \\ 0 & 0 & \boldsymbol{K}_{sb} & \boldsymbol{K}_{ss} \end{bmatrix} \tag{S3}$$

which is an extended version of Equation (16) in the main text. Here Φ, $\boldsymbol{U}$, $\boldsymbol{M}$, and $\boldsymbol{K}$ are displacement potential, displacement, mass matrix, and stiffness matrix. The subscripts f, b, and s represent the fluid domain, solid-fluid coupling boundary, and the solid domain, respectively.

Next, we can construct the global system

$$\boldsymbol{M}^g\ddot{\boldsymbol{U}} = -\boldsymbol{K}^g\boldsymbol{U}, \tag{S4}$$

We observe that the symmetric positive global mass matrix has been altered by the solid-fluid coupling boundary, with the vector $\boldsymbol{A}^T$, and the symmetric global stiffness matrix is also modified by the vector $\boldsymbol{A}$.

Then, we can rewrite the solved system as below:

$$-(\boldsymbol{U}^{n+1} - 2\boldsymbol{U}^n + \boldsymbol{U}^n) = \underbrace{\Delta t^2(\boldsymbol{M}^g)^{-1}\boldsymbol{K}^g}_{\boldsymbol{B}}\boldsymbol{U}^n. \tag{S5}$$

The left side of equation S5 in the frequency domain

$$-(e^{iw\Delta t} - 2 + e^{iw\Delta t})\tilde{\boldsymbol{U}} = 4sin^2(w\Delta t/2)\tilde{\boldsymbol{U}}. \tag{S6}$$

Here, $\tilde{\boldsymbol{U}} = F^+(\boldsymbol{U}(t))$, where F^+ denotes the forward Fourier transform. The coefficient range of $\tilde{\boldsymbol{U}}$ should lie within [0, 4]. The matrix $\boldsymbol{B}$ must be a semi-positive definite matrix, like the updated matrix in FD and SEM, and should only have non-negative real eigenvalues, not greater than 4, to ensure the system's stability (Gaffar and Jiao, 2014; Gao et al., 2018; Lyu et al., 2021).

Since the inverse of the mass matrix exists in this solution system, it is challenging to directly prove theoretically that matrix $\boldsymbol{B}$ is a semi-positive definite matrix. However, we can obtain matrix B directly through an implicit method (by assigning a value of 1 to each degree of freedom one by one, while assigning 0 to the other degrees of freedom). We tested many different solid-fluid coupling models, and all the eigenvalues of the matrix $\boldsymbol{B}$ are non-negative real eigenvalues. These numerical tests demonstrate that the solid-fluid coupling system is stable.

Here, we provide a 2D solid-fluid coupling numerical simulation and list the corresponding eigenvalues. This 2D model is configured as follows: two elements are positioned above and below the CMB, which serves as the common boundary between them. Each element contains 25 GLL points, resulting in a 75 by 75 matrix $\boldsymbol{B}$. The parameters for the lower mantle are Vp = 13,716.62 m/s; Vs = 7,264.65 m/s; and the density = 5,566.46 kg/m^3. For the fluid outer core, Vp = 8,064.79 m/s; and the density = 9,903.44 kg/m^3. The horizontal length is set to 0.125 degrees, and the vertical thickness is 10 km for both the mantle and the outer core. A time step $\Delta t = 0.04\ s$ is used. The final 75 eigenvalues of matrix $\boldsymbol{B}$ are listed in Supplementary Table 1.

Supplementary Table 1: Eigenvalues values of solid-fluid coupling case

1.80560155	1.79540289	1.47762627	1.38795924	1.31430669	1.25104020	1.18298202
1.09122861	1.03591490	1.03439167	0.98351258	0.96965426	0.89059843	0.90386108
0.82284743	0.74462775	0.71307555	0.55803308	0.53830850	0.51723127	0.49315097
0.47443151	0.48120363	0.43209368	0.41603512	0.39537551	0.37825391	0.36374214
0.35286392	0.33648062	0.33494694	0.31647420	0.30962178	0.29282403	0.28083165
0.25563825	0.25070701	0.24295971	0.20265496	0.19607878	0.19325985	0.18488825
0.19064482	0.17191480	0.17525808	0.15843728	0.15405973	0.13217768	0.12055460
0.11061826	0.00102541	0.00813293	0.00750617	0.01560115	0.01884790	0.02010341
0.02256902	0.0278128	0.03126607	0.03564728	0.04111240	0.04608376	0.05412604
0.05731008	0.09605932	0.09327661	0.08869859	0.06577418	0.08339172	0.07063268
0.07204651	0.07809172	0.00000000	0.00000000	0.00000000		

Following the approach introduced by Masson et al., (2014), and based on the representation theorems, the hybrid input mirror forces are constructed using a discrete spatial window function and the discrete wave equation. In what follows, this method is referred to as "YM". In this study, we go a step further and extend the YM method to the case of including solid-fluid coupling. In this case, we need to define two local recovery domains $\Omega_s^{hi} = (\Omega_s^v \cup E_2 \cup E_1)$ and $\Omega_f^{hi} = (\Omega_f^v \cup A_2 \cup A_1)$ (Figure 2) and two window functions:

$$\mathrm{w}_s^{hi}(\mathbf{x}) = \begin{cases} 1, & \text{if } \mathbf{x} \in \Omega_s^{hi} \\ 0, & \text{if } \mathbf{x} \notin \Omega_s^{hi} \end{cases}, \tag{S7}$$

and

$$\mathrm{w}_f^{hi}(\mathbf{x}) = \begin{cases} 1, & \text{if } \mathbf{x} \in \Omega_f^{hi} \\ 0, & \text{if } \mathbf{x} \notin \Omega_f^{hi} \end{cases}. \tag{S8}$$

Then, our primary objective is to derive the explicit mathematical expression for the hybrid input mirror forces at each GLL point of A_1 and E_1. These hybrid input mirror forces will allow us to accurately reconstruct the solid and fluid wavefields within Ω_s^{hi} and Ω_f^{hi} two domains, with the aid of the two window functions $\mathrm{w}_s^{hi}(\mathbf{x})$ and $\mathrm{w}_f^{hi}(\mathbf{x})$.

In the local solid domain Ω_s^{hi} where we want to recover the wavefield, the local displacement wavefield can be written as $\mathbf{u}^{hi}(\mathbf{x}) = \mathbf{u}^g(\mathbf{x})\mathrm{w}_s^{hi}(\mathbf{x})$. Similarly, in the local fluid domain Ω_f^{hi}, the local fluid displacement potential wavefield is expressed as $\varphi^{hi}(\mathbf{x}) = \varphi^g(\mathbf{x})\mathrm{w}_f^{hi}(\mathbf{x})$. During the numerical implementation, we need to reconstruct the discrete approximation of the local solid and fluid wavefield, denoted as $\mathbf{W}_s^{hi}\mathbf{U}$ and $\mathbf{W}_f^{hi}\mathbf{\Phi}$, respectively. Here, $\mathbf{W}_s^{hi}$ and $\mathbf{W}_f^{hi}$ are diagonal matrices corresponding to the values of the window functions $\mathrm{w}_s^{hi}(\mathbf{x})$ and $w_f^{hi}(\mathbf{x})$ evaluated at the Gauss-Lobatto-Legendre (GLL) points. These values are equal to one within the local recovery domains (Ω_s^{hi} and Ω_f^{hi}) and zero outside it. To recover the local wavefield, we can directly generate the hybrid input mirror forces $\mathbf{F}_s^{hi}$ and $\mathbf{F}_f^{hi}$ that fulfill the following conditions.

$$\begin{aligned} \mathbf{M}_s(\mathbf{W}_s^{hi}\ddot{\mathbf{U}}) + \mathbf{K}_s(\mathbf{W}_s^{hi}\mathbf{U}) + \mathbf{A}(\mathbf{W}_f^{hi}[\ddot{\mathbf{\Phi}}]_{fs}) &= \mathbf{F}_s^{hi} \\ \mathbf{M}_f(\mathbf{W}_f^{i}\ddot{\mathbf{\Phi}}) + \mathbf{K}_f(\mathbf{W}_f^{hi}\mathbf{\Phi}) - \mathbf{A}^T(\mathbf{W}_s^{hi}[\mathrm{U}]_{sf}) &= \mathbf{F}_f^{hi}. \end{aligned} \tag{S9}$$

Here we have ignored the absorption boundary matrix because the absorption boundary conditions only apply to the regions where the window functions $\mathrm{w}_s^{hi}(\mathbf{x}) = 0$ and $\mathrm{w}_f^{hi}(\mathbf{x}) = 0$. Based on Equation 16, we observe that the acceleration terms can be expressed in terms of the displacement:

$$\begin{aligned} \ddot{\mathbf{U}} &= \mathbf{M}_s^{-1}(\mathbf{F}_s - \mathbf{C}_s\dot{\mathbf{U}} - \mathbf{K}_s\mathbf{U} - \mathbf{A}[\ddot{\mathbf{\Phi}}]_{fs}) \\ \ddot{\mathbf{\Phi}} &= \mathbf{M}_f^{-1}(\mathbf{0} - \mathbf{C}_f\dot{\mathbf{\Phi}} - \mathbf{K}_f\mathbf{\Phi} + \mathbf{A}^T[\mathrm{U}]_{sf}). \end{aligned} \tag{S10}$$

Next, by substituting the acceleration terms from equation S10 into equation S9, we can derive the hybrid input mirror forces required for the HSFC simulation:

$$\begin{aligned} \mathbf{F}_s^{hi} &= -\mathbf{W}_s^{hi}(\mathbf{K}_s\mathbf{U}) + \mathbf{K}_s(\mathbf{W}_s^{hi}\mathbf{U}) \\ \mathbf{F}_f^{hi} &= -\mathbf{W}_f^{hi}(\mathbf{K}_f\mathbf{\Phi}) + \mathbf{K}_f(\mathbf{W}_f^{hi}\mathbf{\Phi}). \end{aligned} \tag{S11}$$

Upon analyzing the derivation process of equation S11, it becomes apparent that there is no need to explicitly record the physical information on the solid-fluid coupling interface during the global simulation. This is because the calculations with operation [] automatically cancel out. Additionally, note that here the remote seismic source is positioned outside the box, and the contribution of the external source becomes zero.

From equation S11, the numerical hybrid input mirror forces $\mathbf{F}_s^{hi}$ and $\mathbf{F}_f^{hi}$ depend solely on the elements, where the discrete window function $\mathbf{W}_s^{hi}$ and $\mathbf{W}_f^{hi}$ evaluated at the GLL points are not constant and are denoted by E_1 and A_1. When these hybrid input mirror forces are numerically applied to the E_1 and A_1, the equation S11 adds the stored displacement and displacement potential first. Subsequently, the internal force (e.g. $\mathbf{K}_s\mathbf{U}$ in the solid domain) is computed, then the stored solid and fluid internal forces are subtracted. This process follows the approach presented by Masson et al., (2014) and Lyu et al., (2022) for purely elastic and acoustic derivation. Note that in scenarios where the local meshing differs from the corresponding global meshing, spatial interpolation is employed to obtain the displacements/potentials of all GLL points required in the hybrid elements during the global simulations performed by the global solver, such as the package SPECFEM3D_GLOBE.

In the framework of Box Tomography, the structural assumption of the external domain Ω^e remains unchanged, so Green's functions from the remote receiver to the mirror points E_2 and A_2 remain invariant. Consequently, to calculate the outgoing wave propagation from the boundary of the box to the remote receiver, we only need to compute the equivalent body forces at the boundary of the box and the corresponding Green's function from the remote receiver to the boundary of the box, and then convolve them. This transformation allows us to simplify the propagation process of scattering wavefields generated by local anomalies within the box back to distant receivers into a convolution between equivalent body forces and Green's functions. Note that this implementation is similar to the Kirchhoff theory in Wen et al., (1998) with both global and local numerical simulations. However, it is different from studies by Lin et al., (2019) and Leng et al., (2020), where the scattered wave propagates from the boundary of the close box to the remote receiver, using the global numerical simulation not Green's functions. At each GLL point of E_2 and A_2 at the boundary of the box, we can compute the equivalent body forces using the complete wavefields. Note that here we effectively impose the hybrid output mirror forces into E_2 and A_2, to accurately restore the scattered wavefield in the domains $\Omega_s^{he} = (\Omega_s^e \cup \Omega_s^a \cup E_1 \cup E_2)$

and $\Omega_f^{he} = (\Omega_f^e \cup \Omega_f^a \cup A_1 \cup A_2)$. Similarly, at the same GLL points of E_2 and A_2, we can excite a delta source at the remote receiver R^e to obtain Green's functions.

Thus, our second objective is to obtain the explicit mathematical expression of the hybrid output mirror forces, which can accurately restore the solid and fluid wavefields inside two domains Ω_s^{he} and Ω_f^{he} (Figure 2), with the aid of two window functions.

$$\mathrm{w}_s^e(\mathbf{x}) = \begin{cases} 1, & \text{if } \mathbf{x} \in \Omega_s^{he} \\ 0, & \text{if } \mathbf{x} \notin \Omega_s^{he} \end{cases} \tag{S12}$$

and

$$\mathrm{w}_f^e(\mathbf{x}) = \begin{cases} 1, & \text{if } \mathbf{x} \in \Omega_f^{he} \\ 0, & \text{if } \mathbf{x} \notin \Omega_f^{he} \end{cases}. \tag{S13}$$

The window functions $\mathrm{w}_s^{he}(\mathbf{x})$ and $\mathrm{w}_f^{he}(\mathbf{x})$ are utilized to fully reconstruct the wavefield within Ω_s^{he} and Ω_f^{he} while ensuring that the wavefield inside the inversion domain Ω^v is zero-value, denoted as $\mathbf{W}_s^{he}\mathbf{U}$ and $\mathbf{W}_f^{he}\mathbf{\Phi}$. Similar to the method of obtaining the hybrid input mirror forces in equation S11, hybrid output mirror forces $\mathbf{F}_s^{he}$ and $\mathbf{F}_f^{he}$ required for the propagation from the E_2 and A_2 to the remote receiver R^e are mathematically expressed as follows:

$$\begin{aligned} \mathbf{F}_s^{he} &= -\mathbf{W}_s^{he}(\mathbf{K}_s\mathbf{U}) + \mathbf{K}_s(\mathbf{W}_s^{he}\mathbf{U}) \\ \mathbf{F}_f^{he} &= -\mathbf{W}_f^{he}(\mathbf{K}_f\ddot{\mathbf{\Phi}}) + \mathbf{K}_f(\mathbf{W}_f^{he}\ddot{\mathbf{\Phi}}). \end{aligned} \tag{S14}$$

Note that the acceleration potential within the fluid domain is essential for calculating the output mirror forces, distinct from the hybrid input mirror forces. Then, we depend on dimensional analysis to elucidate the reasons for this distinction, aided by the diverse units of physical quantities outlined in Table 1.

The stored hybrid output mirror forces are denoted by the force vector $\mathbf{F}_s^{he}$, measured in physical unit Newtons (N) in an elastic medium. For instance, the unit of the term $\int_{\Omega_s} \boldsymbol{w}\rho_s\ddot{\mathbf{u}}_s d\Omega_s$ is $\frac{kg}{m^3} \cdot \frac{m}{s^2} \cdot m^3 = N$. Note that the test functions are always dimensionless. Green's function is stored with the unit of $\frac{m}{N}$ (meter per Newton). Dividing by Newton N is the definition of Green's function based on unit forces. Therefore, the convolution of forces and Green's functions yields a result with the unit of meters ($N \times \frac{m}{N}$), which corresponds to the required unit of displacement.

Similarly, in a fluid medium, the unit of the term $\int_{\Omega_f} w\frac{1}{\kappa}\ddot{\varphi}d\Omega_f$ is $\frac{m^2}{N}\cdot\frac{N}{m^2}\times m^3 = m^3$ (cubic meter). To generate displacement through convolution between the stored hybrid output mirror forces $\mathbf{F}_f^e$ and the Green's function calculated by global simulation from the remote solid receiver side, the save Green's function should have the unit of $\frac{N}{m^2}$ (Newton per square meter) because $m^3\cdot\frac{\frac{N}{m^2}}{N} = m$. We know the unit of $\ddot{\varphi}$ is $\frac{N}{m^2}$ as shown in Table 1. Hence, if we use φ to calculate the hybrid output mirror forces in the acoustic domain, as in equation S11, we need to record the acceleration potential in the global simulation. Alternatively, we can also use $\ddot{\varphi}$ to calculate the hybrid output forces as in equation S14, in which case the global simulation from the remote receiver side would store φ. Through testing, we have confirmed that these two ways produce identical convolved waveforms.

2 2D hybrid solid-fluid coupling simulation at the Core Mantle Boundary

In the 2D HSFC case at the CMB, the global reference model and the associated Z-wavefield from the remote source S^e side are displayed at time 80 s (Figure S1). The two Z-wavefields of global simulations from the remote receiver R^e side with a delta source time function in the X and Z-directions in the global reference model are illustrated in Figure S2.

In the 2D SORI case, when the local model is the same as the global reference model, and the local mesh matches the global mesh, the waveforms obtained from the hybrid simulation are almost identical to those obtained from the global simulation (Figure S3ab). This example demonstrates the high accuracy of the HSFC simulation, making it a highly reliable forward modeling tool for Box Tomography. It lays a theoretical foundation for the future practical applications of Box Tomography containing a solid-fluid coupling interface in the SORI case. Similarly, Figure S3cdef displays the waveforms benchmark between the global and hybrid simulations in the global and two different local target models, for the same receiver R^i inside the box. For the local target model with a ULVZ above CMB, the relative errors of the Ux and Uz components are about 1.5% and 0.5%, respectively. For the local target model with an undulating CMB, the relative errors of the Ux and Uz components are about 2.68% and 1.25%. Despite the overall similarity between the two waveforms, the imperfections of the absorbing boundaries contribute

to this minor discrepancy. The blue vertical dashed lines (Figure S3) represent the moment of the wavefield (Figure S1b).

3 2D hybrid solid-fluid coupling with the Ocean, Crust, and Mantle

For the 2D HSFC case near the seafloor, the global reference model and the associated Z-wavefield from the source side are presented in Figure S4. Figure S5 shows the Z-wavefields of two global simulations from the remote receiver R^e side also in the global reference model.

4 3D hybrid solid-fluid coupling

In the 3D HSFC case, the source time function used is a Heaviside wavelet with four cut-off filtered periods of 15, 20, 150, and 200 s. The simulated duration is 2400 s. The remote source is positioned at a depth of 35 km beneath the surface with latitude and longitude (50°, -22.5°). Similar to the 2D case, two receivers are considered. The first one is located inside the box at a depth of 250 km right above the CMB with coordinates at (90°, 0°, 3730 km), represented as a triangle (Figure 8a). The second receiver is located outside the domain with coordinates on the surface at (50°, 157.5°), the same latitude as the source. The local model has dimensions of (40°, 40°, 1700 km) with 1000 km in the lower mantle and 700 km in the outer core. We spatially discretize it into a mesh consisting of 54×54×(16+15) elements with NGLL=5. The local ULVZ has dimensions of approximately 200 km×50 km, with reductions of 43% in S-wave velocity, 21.5% in P-wave velocity, and 14.3% in density compared to the surrounding medium. While the density of real ULVZs might show a positive perturbation, we have chosen to follow the fixed scaling used in FWI: $\frac{\ln V_p}{\ln V_s} = \frac{1}{2}$ and $\frac{\ln V_p}{\ln \rho} = \frac{3}{2}$. The time step $\Delta t \approx$0.0855 s in the global simulations and $\Delta t =$0.3 s in the local simulations.

We use the SPECFEM3D_GLOBE code for the global simulation, with selected parameters NEX_XI=640 and NPROC_XI=16, on ANVIL (Song et al., 2022). Using 1536 CPUs, it takes about 7.72 hours to finish the calculation. The global mesh contains 55,539,200 spectral elements, and the local mesh comprises 90,396 spectral elements.

Compared to the scattered phase due to the local anomaly (Figure 7), there is no scattered wave propagating outside the box when the local model matches the global model (Figure S6).

We then present the 3D SORI and SORO cases to show the accuracy of the hybrid waveforms with the N and Z components in Figure S7 and Figure S8

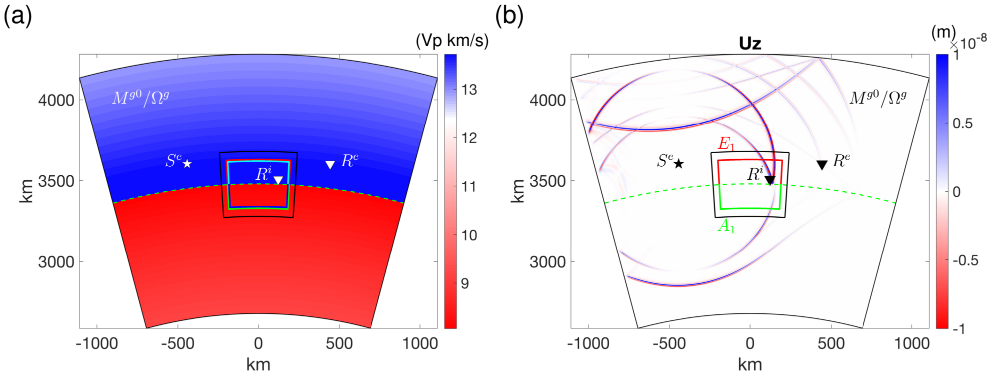

Figure S1: (a) The 2D Global Reference Model M^{g0} containing the Core-Mantle Boundary. The size of the global domain Ω is (30°, 1600 km) laterally, and extends 800 km both above and below the CMB. The remote source is a Ricker wavelet source, with dominant and maximum frequencies of f_0 = 0.6 Hz and $f_{\text{max}} \approx 2.5f_0$ = 1.5 Hz, respectively. A structured mesh is employed for both the global and local simulations, with the global mesh comprising 480×(160+160) elements and 5 GLL points (NGLL = 5) used in the spectral element computation. The source S^e is located at (-7°, 3630 km), 150km above CMB, where the angle is defined with the z-axis. Two receivers are placed: one R^i inside the box located at (-1.5°, 3510 km), 30km above the CMB and another R^e outside the box located at (7°, 3630 km). The global simulation involves a free surface, and 10 elements were used for the absorbing boundary condition near the left, right, and bottom boundaries. (b) The associated global simulations from the remote source side. The star symbolizes the remote source, while the two inverted triangles represent two receivers located inside and outside the enclosed local box (depicted in black). In this global numerical simulation from the remote source side, the hybrid input mirror forces are calculated and stored at the Gauss-Lobatto-Legendre (GLL) points at mirror E_1 (red) within the local elastic domain and A_1 (green) within the local acoustic domain.

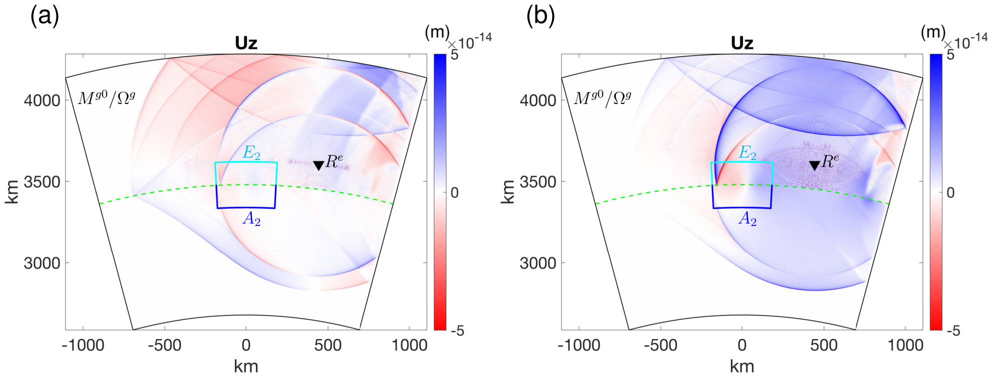

Figure S2: Two 2D global simulations from the remote receiver R^e side carried out in the reference model M^{g0} containing the Core Mantle Boundary to derive the Green's functions at GLL points of mirror E_2 (cyan) within the local elastic domain and mirror A_2 (blue) within the local acoustic domain. These simulations were conducted utilizing a delta source in time at the location of the remote receiver R^e (reversed triangle), with a single force applied in the x-axis (a) and the z-axis (b) directions, respectively.

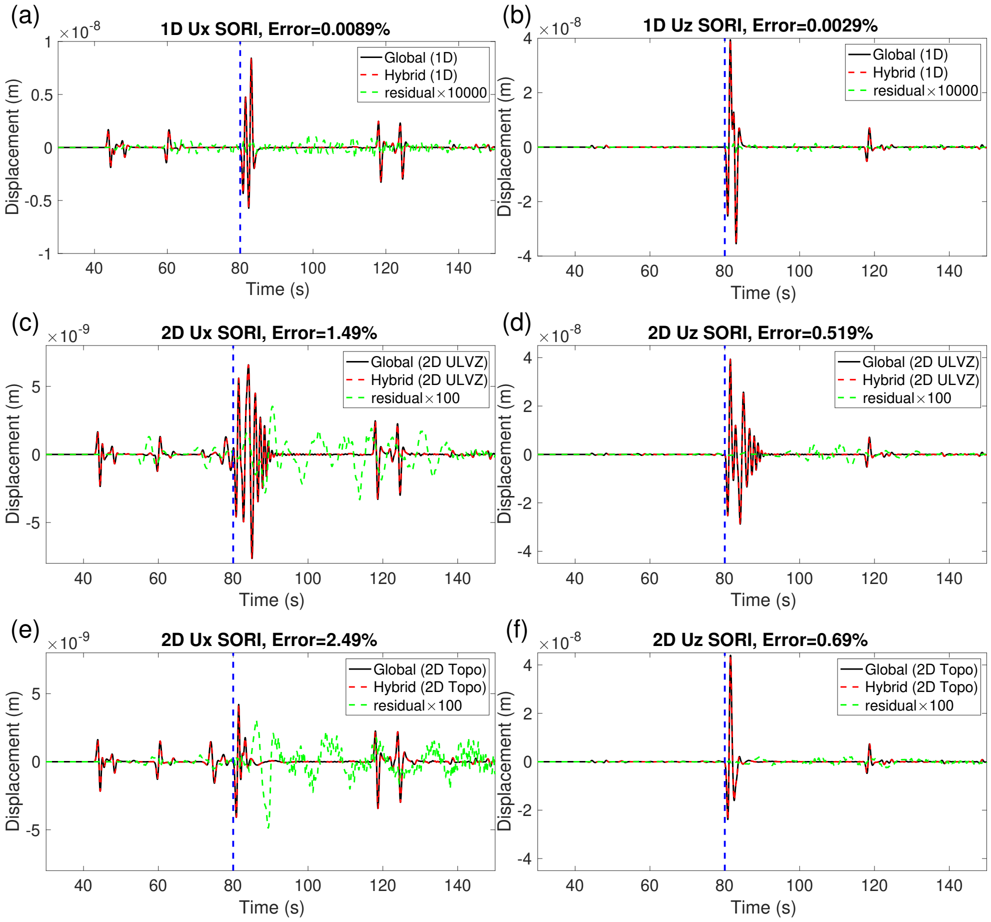

Figure S3: Waveform benchmarks for the SORI case, focusing on HSFC near the Core Mantle Boundary (CMB). (a,b) X and Z component Waveforms at receiver R^i inside the enclosed box under both the global and local reference models. (c,d) X and Z component waveforms at the same receiver for both the global and local target models with an Ultra-Low Velocity Zone (ULVZ) above CMB. (e,f) X and Z component waveforms at the same receiver for both the global and local target models, considering an undulating CMB. The solid black lines correspond to data obtained from global simulations in the global models. The dashed red lines represent results from hybrid simulations in the local models. The dashed green lines depict the residuals, magnified by different factors.

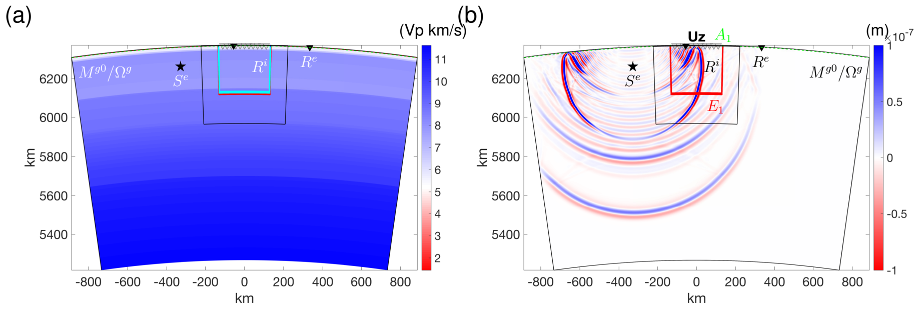

Figure S4: (a) The 2D global reference model M^{g0} containing the ocean, crust and mantle. The star symbolizes the remote source, while the two black inverted triangles represent two receivers located inside and outside the enclosed local box, and 11 receivers (gray triangles) are located inside the ocean part of the local model. The dimensions of the global domain Ω^g are (16°, 1100 km), encompassing a 3 km ocean region. The local domain, enclosed within the black box has dimensions of (4°, 400 km), which also includes the 3 km ocean. For both global and local numerical simulations, the remote source is a Ricker wavelet. The source S^e is located at (-3°, 6271 km), 100 km beneath the free surface, with the angle defined relative to the z-axis. One solid receiver R^i is placed inside the box located at (-0.5°, 6368 km), situated at the ocean bottom, and another solid receiver R^e is outside the box located at (+3°, 6368 km), also at the ocean bottom. We record displacement at these two solid stations. Eleven additional receivers are positioned at intervals of 0.2° within the ocean, ranging from -1° to +1°, at a depth of 1 km beneath the free surface. Pressure is recorded at these eleven stations. The central frequency is set at f_0 = 0.2 Hz with the highest frequency f_{max} around 0.5 Hz. To ensure accurate numerical simulation with a maximum frequency of 0.5 Hz, the global mesh consists of 1024×(1+123) structural elements, while the local mesh contains 256×(1+51) elements, 1 element is used to represent the ocean in the z direction both in the global and local meshes. A time step $\Delta t = 0.01$ s is used in the global simulation. 50 elements are used for the absorbing boundary condition near the left and right boundaries and 20 elements for the bottom boundary, given the extremely low Vp velocity in the ocean (1450 m/s). (b) The associated global simulations from the remote source side. In the global numerical simulation, we calculate and store the hybrid input mirror forces at the GLL points of the mirror E_1 (red) within the elastic domain and mirror A_1 (green, referring to Figure 5a for a locally enlarged version).

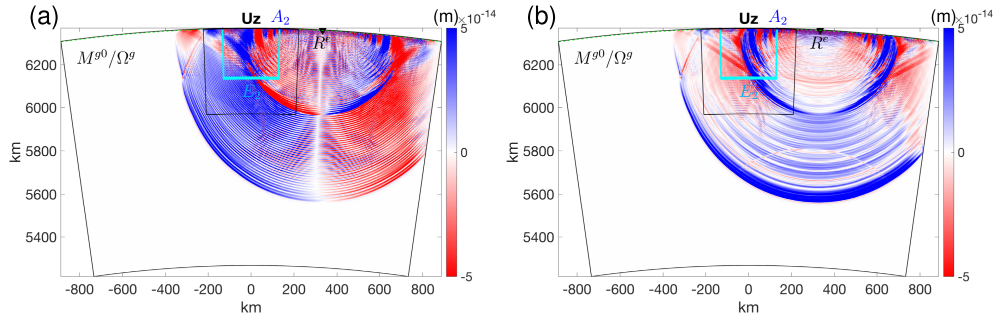

Figure S5: Two 2D global simulations were carried out in the reference model M^{g0} containing the ocean, crust, and mantle to derive the Green's functions at GLL points on mirror E_2 (cyan) within the local elastic domain and mirror A_2 (blue, referring to Figure 5a for a locally enlarged version) within the local acoustic domain. These simulations were conducted utilizing a delta source in time at the location of the remote receiver R^e (depicted by the reversed triangle), with a single force applied in the x-axis (a) and the Z-axis (b) directions, respectively.

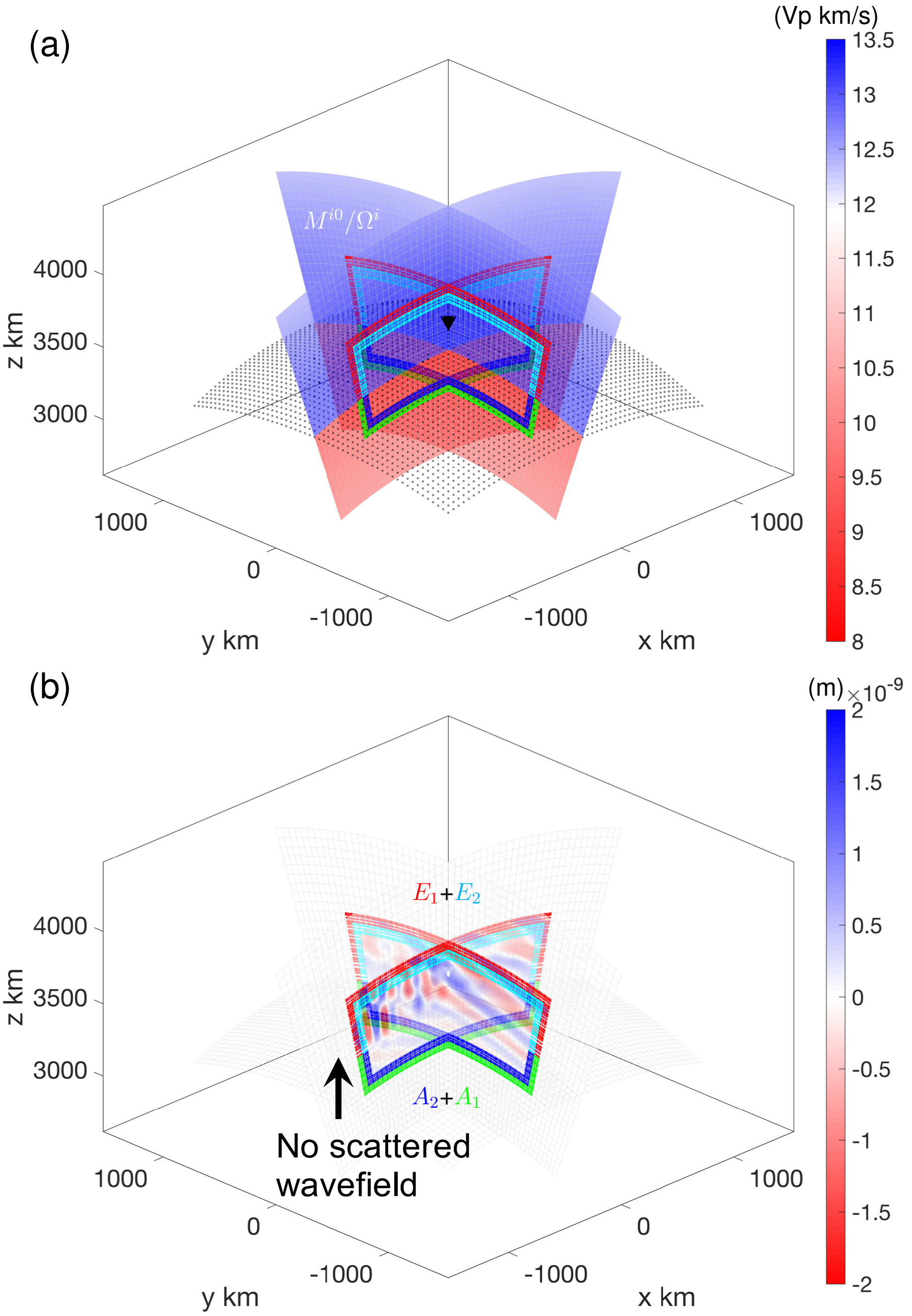

Figure S6: Regional 3D reference model and hybrid wavefield. (a) Reference model consists of the lower mantle of SEMUCB_WM1 and the outer core of PREM. The black dotted surface represents the Core-Mantle Boundary (CMB). (b) Local Z-component Wavefield at 1260 seconds.

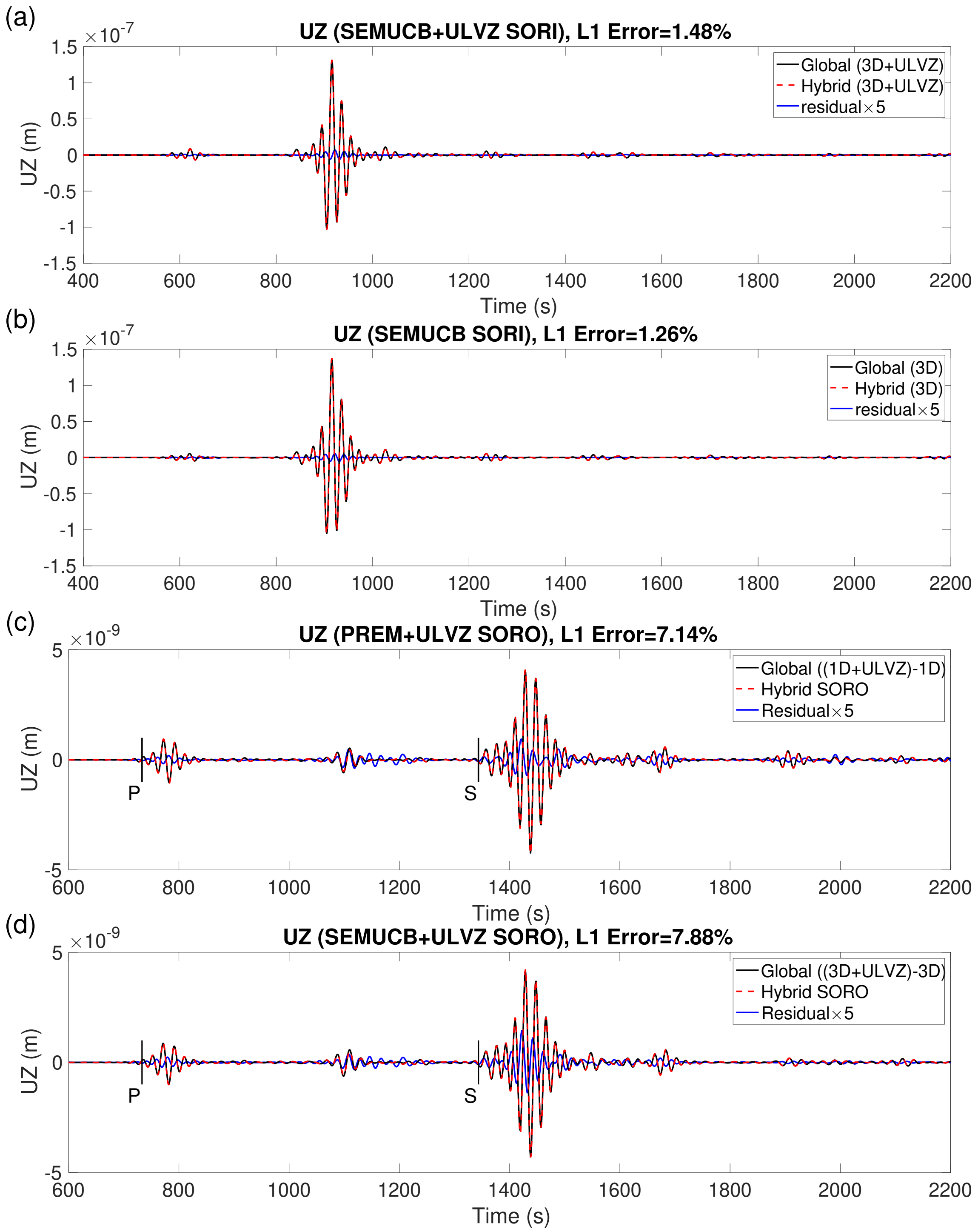

Figure S7: Global and hybrid simulation waveform comparison. Waveforms obtained from global simulations are represented in black, while those from hybrid simulations are depicted in red, with differences highlighted through magnified green residuals by a factor of 5. Z-component waveforms for a receiver inside the box are detailed in (a, b) using the SEMUCB_WM1 reference model and SEMUCB_WM1 with a localized ultra-low velocity zone, respectively. (c, d) Z-component waveforms for a receiver outside the box using reference models PREM and SEMUCB_WM1, respectively, both featuring a localized ultra-low velocity structure.

We then continue to explain the larger error in the SORO case compared to the SORI case in the 3D case. First, the hybrid input mirror forces are obtained in the global simulation from the source side by computing spatial interpolations at the E_1 and A_1 GLL points. Consequently, they carry spatial dispersion errors, which is also the reason for errors in the SORI case. As a result, the resultant hybrid output mirror forces that we obtain also have spatial dispersion errors, inherited from the hybrid input mirror forces. On the other hand, Green's functions are obtained in the global simulation from the receiver side through spatial interpolations at the E_2 and A_2 GLL points, leading to errors in Green's functions as well. Since convolution is a linear operator, the errors in hybrid output mirror forces and Green's functions will linearly superpose, leading to an increase in the overall error.

5 Convergence

To validate the convergence of HSFC simulations, we first design a 2D global target model (Figure S9) and a local target model (Figure S10a1).

Then we perform the global and hybrid simulations of localized and smooth identical ULVZ at seven different minimum periods (Figure S10a2-a8). Both the SORI and SORO cases converge (Figure S10bcde).

6 Waveform integrity

To illustrate the delayed and incomplete waveform (second-order scattering) of the HSFC simulation, we rely on the global and local target models used in the preceding convergence section. However, we modify the four boundaries of the global model into free surfaces to enhance the energy of second-order scattering for demonstration purposes, and we perform a relatively long duration (600 s) simulation to show these delayed scattered phases. The resulting waveforms are plotted in Figure S11.

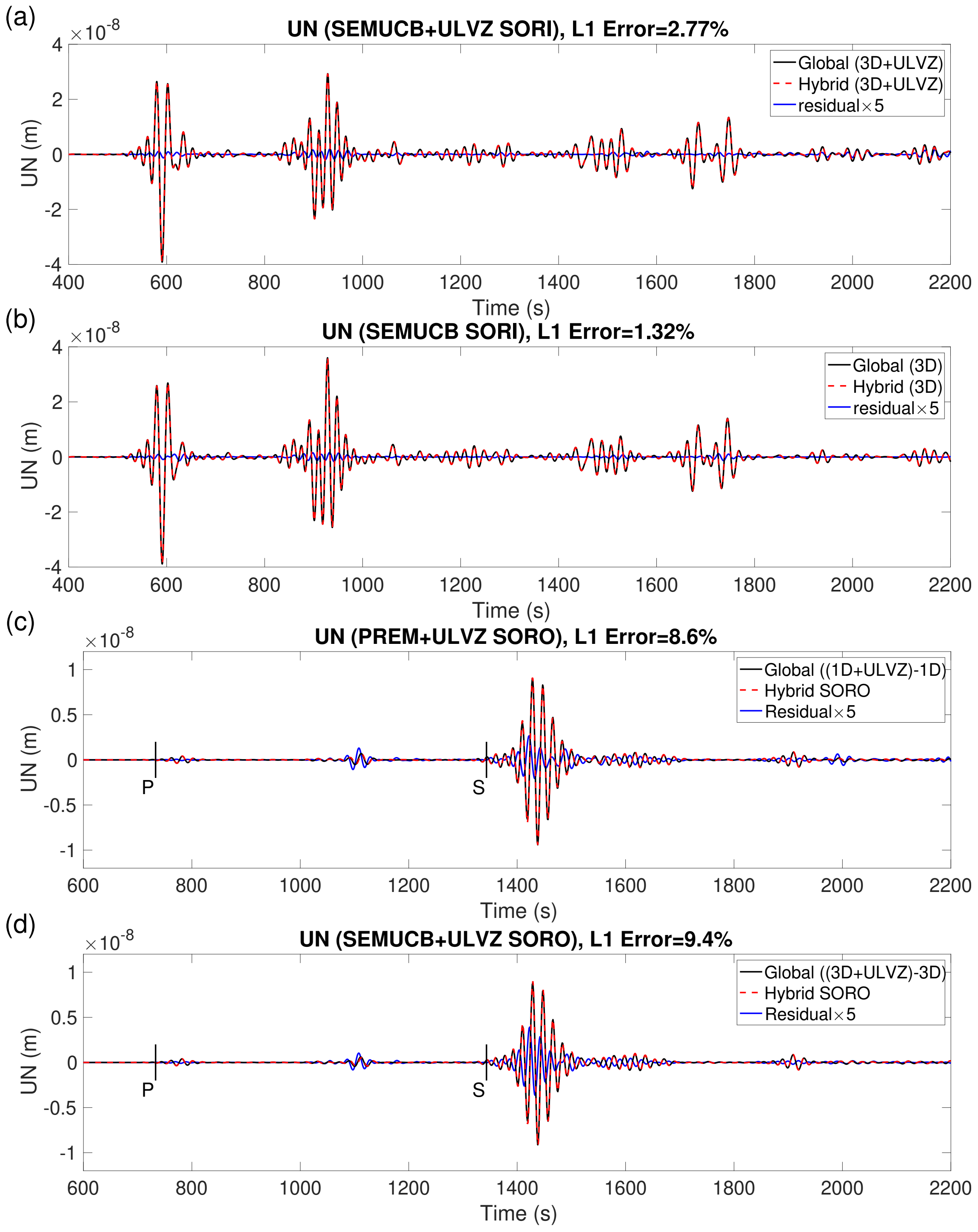

Figure S8: Same as the Figure S7 above, but for the N-component.

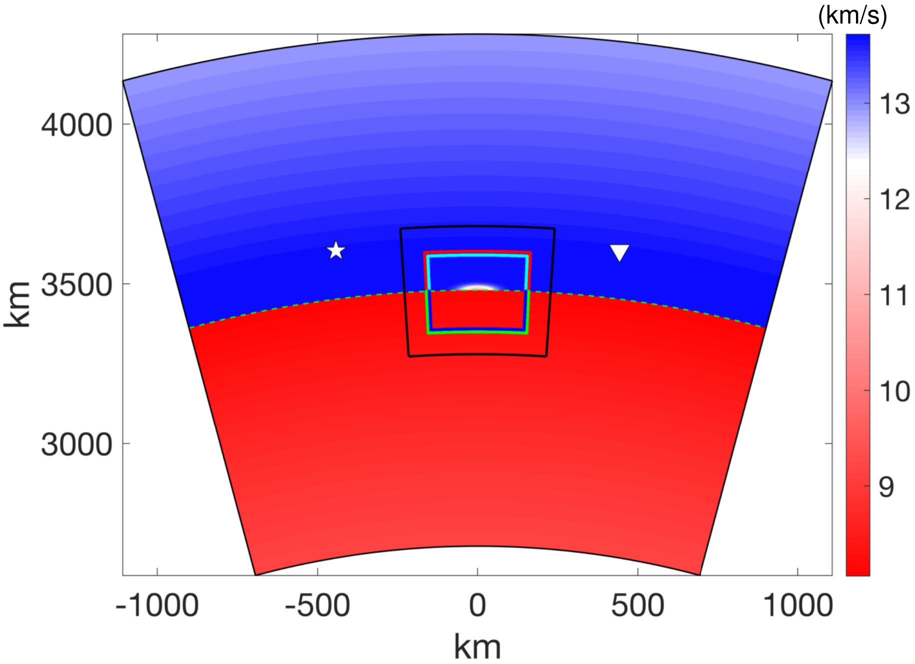

Figure S9: A global 2D target model with a smooth and localized ultra-low velocity zone. The size of the global model is identical to the one shown in Figure S1a.

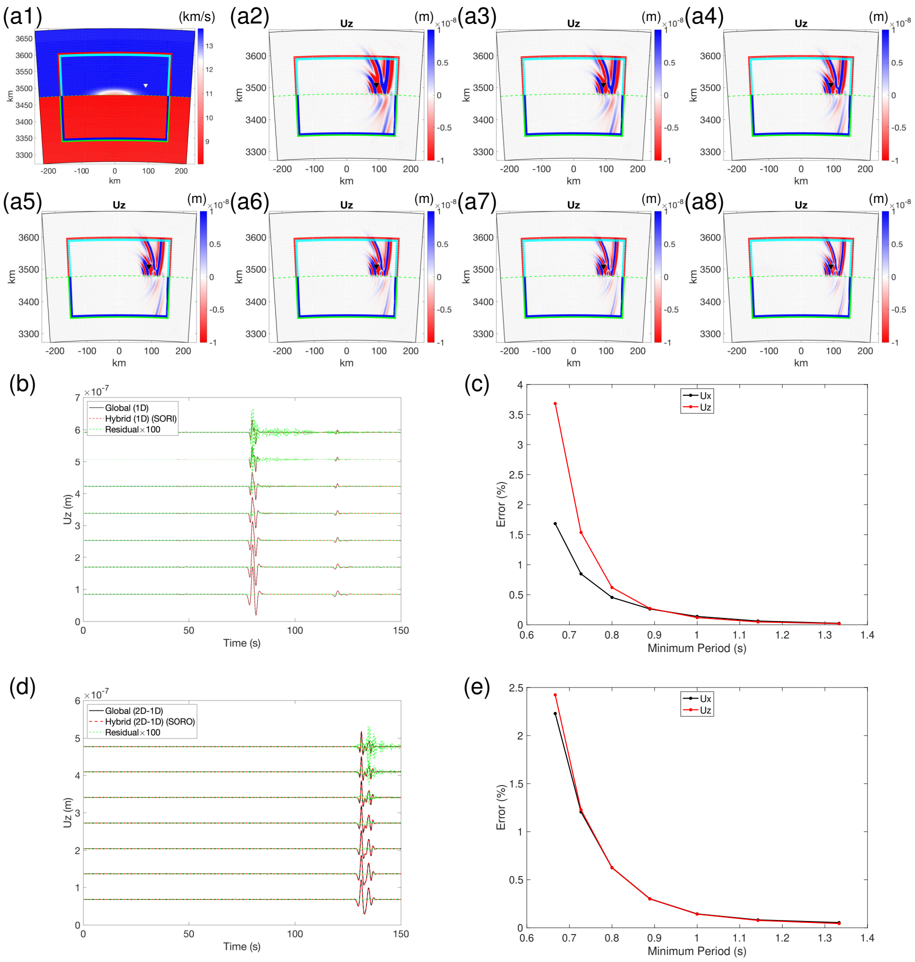

Figure S10: Convergence analysis of 2D wavefields and waveforms in the hybrid solid-fluid coupling in the SORI and SORO cases. For different local and global spatial meshings in testing, we constructed a Gaussian-shaped Ultra-Low-Velocity Zone (ULVZ). The global and local grid meshing was established based on a minimum period of 0.67 seconds. Subsequently, we conducted simulations with seven different minimum periods, associated maximum frequencies ranging from 0.75 Hz, 0.875 Hz, 1.0 Hz, 1.125 Hz, 1.25 Hz, 1.375 Hz, to 1.50 Hz. (a1) illustrates the Vp (P-wave velocity) distribution for the local model. (a2), a(3) to (a8) correspond to the local wavefield for increasing periods from long to short. (b) displays the Z-component waveforms at stations within the box, with the top-to-bottom order representing periods from long to short. (c) depicts the error distribution of waveform X and Z-components. (d) and (e) are similar to (b) and (c) but correspond to stations located outside the box.

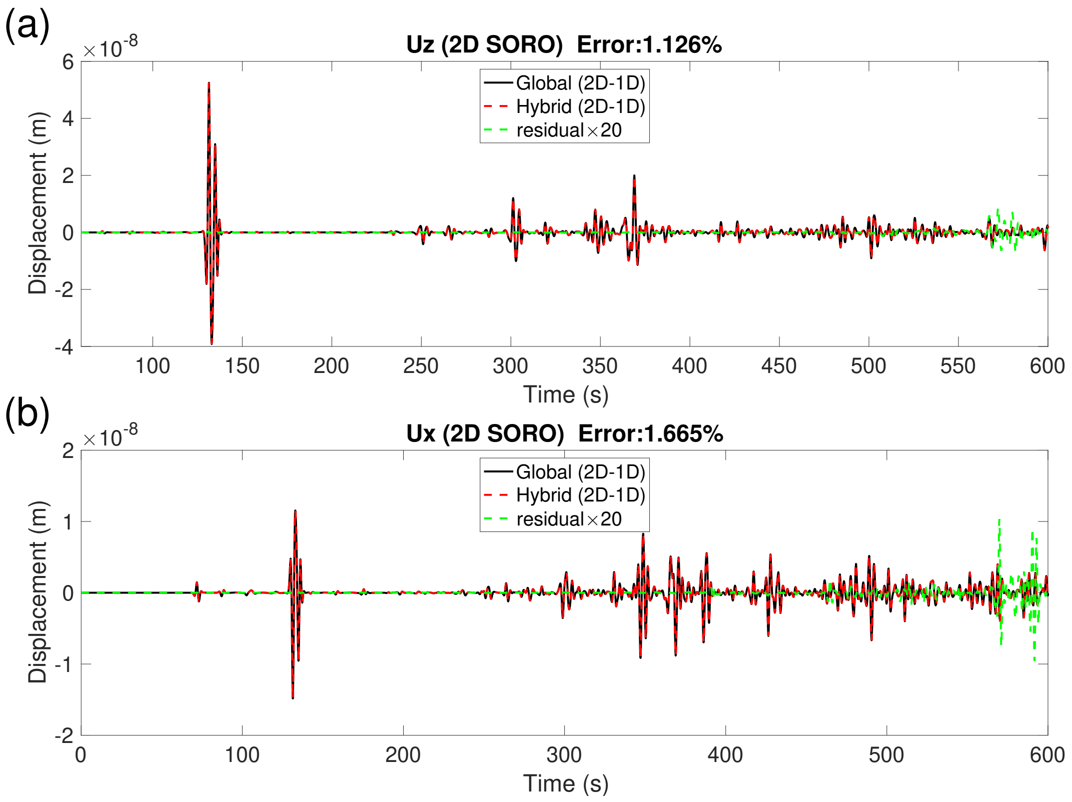

Figure S11: The hybrid X and Z-component waveforms in the SORO case, corresponding to the model in Figure S9.

7 Absorbing boundary layers

When the local model M^{i1} contains anomalies different from the reference local model M^{i0}, scattered solid and fluid waves are generated during the local simulation that contribute to generating the output mirror forces. When convolved with the Green's functions computed in the reference global model on the remote receiver side, this scattered wavefield will propagate outside of the box. To prevent the outgoing scattered waves from returning to the local domain, which would compromise the stability and accuracy of the hybrid simulation, additional absorbing conditions are required at the boundaries of the box.

Although Perfectly Matched Layers (PML) have been successful in many applications, developing a stable formulation for general solid-fluid coupling using the continuous Galerkin spec-

tral element method, remains an open research question. The Clayton-Engquist condition works fairly well for waves with normal incident angles but is not satisfactory for others **?**. Following the work of Kosloff and Kosloff**?** and Yao et al. **?**, we introduce additional sponge layers close to the boundaries within which the wavefield is tapered. Subsequent numerical tests have demonstrated that using a layer width of 9-10 wavelengths with an exponential function usually yields good results and ensures robust stability, particularly in the regional simulation of anisotropic models under the solid-fluid coupling framework. After introducing the sponge layer, equations (13) are modified as follows:

$$\begin{aligned}\int_{\Omega_s} \boldsymbol{w}\rho_s(\ddot{\mathbf{u}}_s + 2\mathbf{D}_s\dot{\mathbf{u}}_s + \mathbf{D}_s^2\mathbf{u}_s)d\Omega_s + \int_{\Omega_s} \nabla\boldsymbol{w} : \boldsymbol{C} : \nabla\boldsymbol{u}d\Omega_s + \int_{\Gamma_c} \boldsymbol{w}\cdot\ddot{\varphi}\mathbf{n}d\Gamma &= \int_{\Omega_s} \boldsymbol{w}\boldsymbol{f}_s d\Omega_s \\ \int_{\Omega_f} w\frac{1}{\kappa}(\ddot{\varphi} + 2D_f\dot{\varphi} + D_f^2\varphi)d\Omega_f + \int_{\Omega_f} \nabla w\cdot(\frac{1}{\rho_f}\nabla\varphi)d\Omega_f - \int_{\Gamma_c} w\mathbf{u}_s\boldsymbol{n}d\Gamma &= 0, \end{aligned} \quad \text{(S15)}$$

where the original $\ddot{\mathbf{u}}_s$ and $\ddot{\varphi}$ in equation (13) have been replaced by $\ddot{\mathbf{u}}_s + 2\mathbf{D}_s\dot{\mathbf{u}}_s + \mathbf{D}_s^2\mathbf{u}_s$ and $\ddot{\varphi} + 2D_f\dot{\varphi} + D_f^2\varphi$. The taper functions $\mathbf{D}_s$ are D_f is defined as functions of distance d from the boundaries as follows:

$$D(d) = \begin{cases} log(\frac{1}{R})\frac{V_p\times d}{l^2},\ 0 \le d \le l \\ 0,\ else. \end{cases} \quad \text{(S16)}$$

The sponge layer is parameterized by the reflection ratio R and the width of the absorbing layer l, where R=0.0001 is used in this study. The second-order central finite difference scheme is applied to the time derivative of the modified equations (S15). To ensure the accuracy of the hybrid simulation of the domain containing the solid-fluid coupling interface, the seismic velocity structures within the mirrors E/A must remain unchanged during the hybrid wavefield simulations.

8 Animations

Here, we have added 23 animations from mov1 to mov23 related to the numerical simulations in the main text.

- Movie 1 (SF2d_global_fullx_homo60.mov): the global wavefield animations of the X-component in the global reference model, from the source side in the 2D HSFC case with CMB.
- Movie 2 (SF2d_global_fullz_homo60.mov): the global wavefield animations of the Z-component in the global reference model, from the source side in the 2D HSFC case with CMB.
- Movie 3 (SF2d_global_fullx_hete60.mov): the global wavefield animations of the X-component in the global target model, from the source side in the 2D HSFC case with CMB.
- Movie 4 (SF2d_global_fullz_hete60.mov): the global wavefield animations of the Z-component in the global target model, from the source side in the 2D HSFC case with CMB.
- Movie 5 (SF2d_global_residualx60.mov): the global residual wavefield of the X-component between SF2d_global_fullx_homo60.mov and SF2d_global_fullx_hete60.mov, from the source side in the 2D HSFC case with CMB.
- Movie 6 (SF2d_global_residualz60.mov): the global residual wavefield of the Z-component between SF2d_global_fullz_homo60.mov and SF2d_global_fullz_hete60.mov, from the source side in the 2D HSFC case with CMB.
- Movie 7 (SF2d_global_greenfx_homo60.mov): the global wavefield animations of the X-component in the global reference model, from the receiver side in the 2D HSFC case with CMB.
- Movie 8 (SF2d_global_greenfz_homo60.mov): the global wavefield animations of the Z-component in the global reference model, from the receiver side in the 2D HSFC case with CMB.
- Movie 9 (SF2d_local_fullz_homo60.mov): the local wavefield animations of the Z-component in the local reference model, corresponding to the maximum frequency of 1.50 Hz in the 2D HSFC case with CMB.

- Movie 10 (SF2d_local_fullz_hete_ulvz60.mov): the local wavefield animations of the Z-component in the local ultra-low velocity (ULVZ) model, corresponding to the maximum frequency of 1.50 Hz in the 2D HSFC case with CMB.

- Movie 11 (SF2d_local_fullz_hete_topo60.mov): the local wavefield animations of the Z-component in the local undulating CMB model, corresponding to the maximum frequency of 1.50 Hz in the 2D HSFC case with CMB.

- Movie 12 (SF2d_local_residualz_ulvz60.mov): the local residual wavefield animations of the Z-component between the local reference and ULVZ model, corresponding to the maximum frequency of 1.50 Hz in the 2D HSFC case with CMB.

- Movie 13 (SF2d_local_residualz_hete_topo60.mov): the local residual wavefield animations of the X-component between the local reference and undulating CMB model, corresponding to the maximum frequency of 1.50 Hz in the 2D HSFC case with CMB.

- Movie 14 (SF3d_local_fullz_homo.mov): the 3D local wavefield animations of the Z-component in the global reference model, from the source side.

- Movie 15 (SF3d_local_fullz_hete.mov): the 3D local wavefield animations of the Z-component in the global target model, from the source side.

- Movie 16 (SF3d_local_residualz.mov): the 3D local residual wavefield of the Z-component between SF3d_local_fullx_homo.mov and SF3d_local_fullx_hete.mov, from the source side.

- Movie 17 (SF2d_local_fullz_hete30_same.mov): the local wavefield animations of the Z-component in the local target model, corresponding to the maximum frequency of 0.750 Hz.

- Movie 18 (SF2d_local_fullz_hete35_same.mov): the local wavefield animations of the Z-component in the local target model, corresponding to the maximum frequency of 0.875 Hz.

- Movie 19 (SF2d_local_fullz_hete40_same.mov): the local wavefield animations of the Z-component in the local target model, corresponding to the maximum frequency of 1.000 Hz.

- Movie 20 (SF2d_local_fullz_hete45_same.mov): the local wavefield animations of the Z-component in the local target model, corresponding to the maximum frequency of 1.125 Hz.

- Movie 21 (SF2d_local_fullz_hete50_same.mov): the local wavefield animations of the Z-component in the local target model, corresponding to the maximum frequency of 1.250 Hz.
- Movie 22 (SF2d_local_fullz_hete55_same.mov): the local wavefield animations of the Z-component in the local target model, corresponding to the maximum frequency of 1.375 Hz.
- Movie 23 (SF2d_local_fullz_hete60_same.mov): the local wavefield animations of the Z-component in the local target model, corresponding to the maximum frequency of 1.500 Hz.
