# Supplementary material for: Efficient hybrid numerical modeling of the seismic wavefield in the presence of solid-fluid boundaries
Source: Nat Commun. 2025 Feb 18;16:1722. doi: 10.1038/s41467-025-56530-5 (PMC11836316; doi:10.1038/s41467-025-56530-5)
Supplement: Supplementary file 2 — Description of Additional Supplementary Files [file 41467_2025_56530_MOESM2_ESM.pdf]

Description of Additional Supplementary Files

Supplementary Movie 1~(SF2d_global_fullx_homo60.mov): the global wavefield animations of the X-component in the global reference model, from the source side in the 2D HSFC case with CMB.

Supplementary Movie 2~(SF2d_global_fullz_homo60.mov): the global wavefield animations of the Z-component in the global reference model, from the source side in the 2D HSFC case with CMB.

Supplementary Movie 3~(SF2d_global_fullx_hete60.mov): the global wavefield animations of the X-component in the global target model, from the source side in the 2D HSFC case with CMB.

Supplementary Movie 4~(SF2d_global_fullz_hete60.mov): the global wavefield animations of the Z-component in the global target model, from the source side in the 2D HSFC case with CMB.

Supplementary Movie 5~(SF2d_global_residualx60.mov): the global residual wavefield of the X-component between SF2d_global_fullx_homo60.mov and SF2d_global_fullx_hete60.mov, from the source side in the 2D HSFC case with CMB.

Supplementary Movie 6~(SF2d_global_residualz60.mov): the global residual wavefield of the Z-component between SF2d_global_fullz_homo60.mov and SF2d_global_fullz_hete60.mov, from the source side in the 2D HSFC case with CMB.

Supplementary Movie 7~(SF2d_global_greenfx_homo60.mov): the global wavefield animations of the X-component in the global reference model, from the receiver side in the 2D HSFC case with CMB.

Supplementary Movie 8~(SF2d_global_greenfz_homo60.mov): the global wavefield animations of the Z-component in the global reference model, from the receiver side in the 2D HSFC case with CMB.

Supplementary Movie 9~(SF2d_local_fullz_homo60.mov): the local wavefield animations of the Z-component in the local reference model, corresponding to the maximum frequency of 1.50~Hz in the 2D HSFC case with CMB.

Supplementary Movie 10~(SF2d_local_fullz_hete_ulvz60.mov): the local wavefield animations of the Z-component in the local ultra-low velocity (ULVZ) model, corresponding to the maximum frequency of 1.50~Hz in the 2D HSFC case with CMB.

Supplementary Movie 11~(SF2d_local_fullz_hete_topo60.mov): the local wavefield animations of the Z-component in the local undulating CMB model, corresponding to the maximum frequency of 1.50~Hz in the 2D HSFC case with CMB.

Supplementary Movie 12~(SF2d_local_residualz_ulvz60.mov): the local residual wavefield animations of the Z-component between the local reference and ULVZ model, corresponding to the maximum frequency of 1.50~Hz in the 2D HSFC case with CMB.

Supplementary Movie 13~(SF2d_local_residualz_hete_topo60.mov): the local residual wavefield animations of the X-component between the local reference and undulating CMB model, corresponding to the maximum frequency of 1.50~Hz in the 2D HSFC case with CMB.

Supplementary Movie 14~(SF3d_local_fullz_homo.mov): the 3D local wavefield animations of the Z-component in the global reference model, from the source side.
Movie 15~(SF3d_local_fullz_hete.mov): the 3D local wavefield animations of the Z-component in the global target model, from the source side.

Supplementary Movie 16~(SF3d_local_residualz.mov): the 3D local residual wavefield of the Z-component between SF3d_local_fullx_homo.mov and SF3d_local_fullx_hete.mov, from the source side.

Supplementary Movie 17~(SF2d_local_fullz_hete30_same.mov): the local wavefield animations of the Z-component in the local target model, corresponding to the maximum frequency of 0.750~Hz.
Supplementary Movie 18~(SF2d_local_fullz_hete35_same.mov): the local wavefield animations of the Z-component in the local target model, corresponding to the maximum frequency of 0.875~Hz.

Supplementary Movie 19~(SF2d_local_fullz_hete40_same.mov): the local wavefield animations of the Z-component in the local target model, corresponding to the maximum frequency of 1.000~Hz.

Supplementary Movie 20~(SF2d_local_fullz_hete45_same.mov): the local wavefield animations of the Z-component in the local target model, corresponding to the maximum frequency of 1.125~Hz.

Supplementary Movie 21~(SF2d_local_fullz_hete50_same.mov): the local wavefield animations of the Z-component in the local target model, corresponding to the maximum frequency of 1.250~Hz.

Supplementary Movie 22~(SF2d_local_fullz_hete55_same.mov): the local wavefield animations of the Z-component in the local target model, corresponding to the maximum frequency of 1.375~Hz.

Supplementary Movie 23~(SF2d_local_fullz_hete60_same.mov): the local wavefield animations of the Z-component in the local target model, corresponding to the maximum frequency of 1.500~Hz.
