# Supplementary figures and images for: Efficient hybrid numerical modeling of the seismic wavefield in the presence of solid-fluid boundaries
Source: Nat Commun. 2025 Feb 18;16:1722. doi: 10.1038/s41467-025-56530-5 (PMC11836316; doi:10.1038/s41467-025-56530-5)

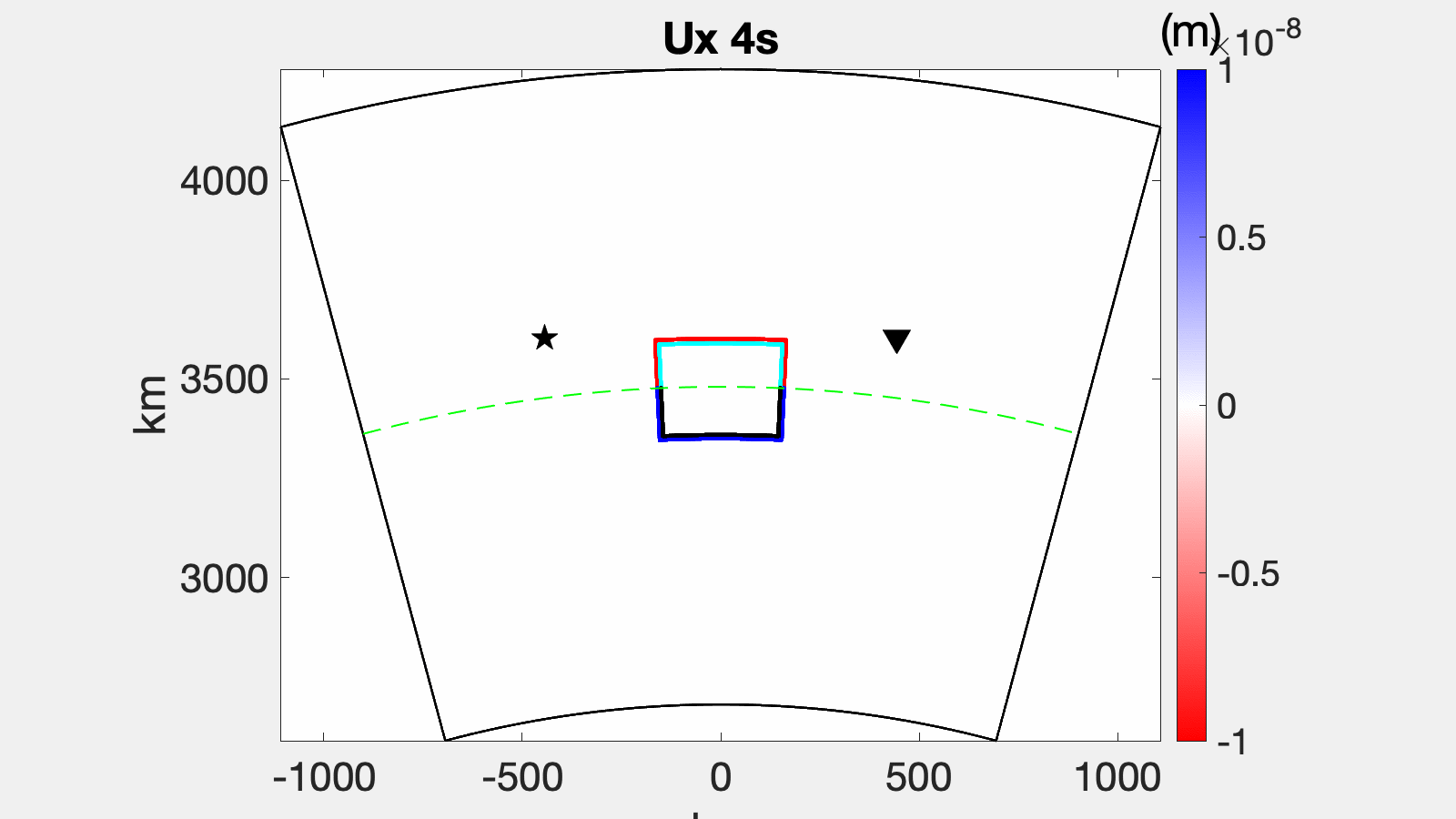

Supplement: Supplementary file 3 — Supplementary Movie 1 [file 41467_2025_56530_MOESM3_ESM.gif]

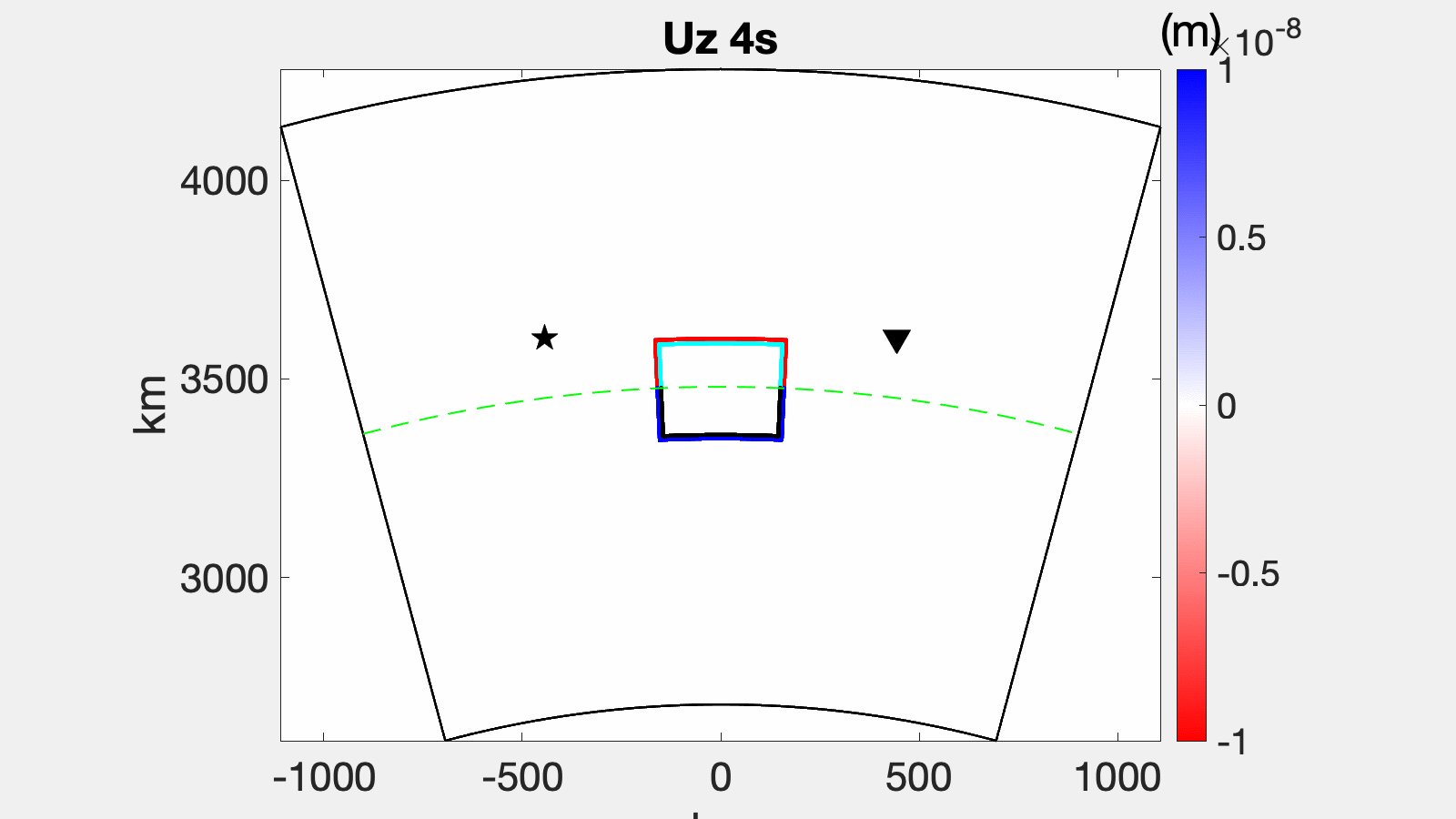

Supplement: Supplementary file 4 — Supplementary Movie 2 [file 41467_2025_56530_MOESM4_ESM.gif]

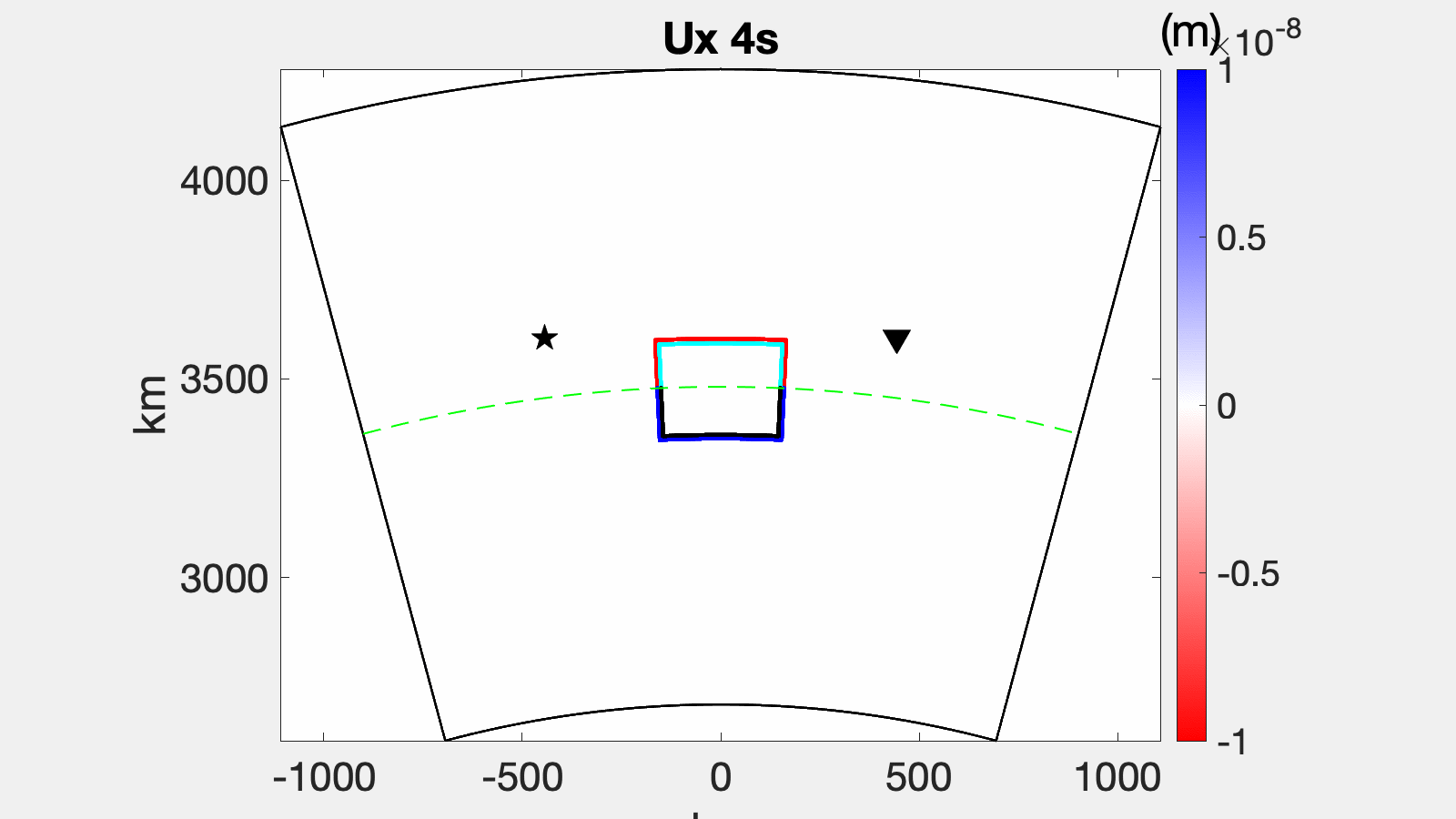

Supplement: Supplementary file 5 — Supplementary Movie 3 [file 41467_2025_56530_MOESM5_ESM.gif]

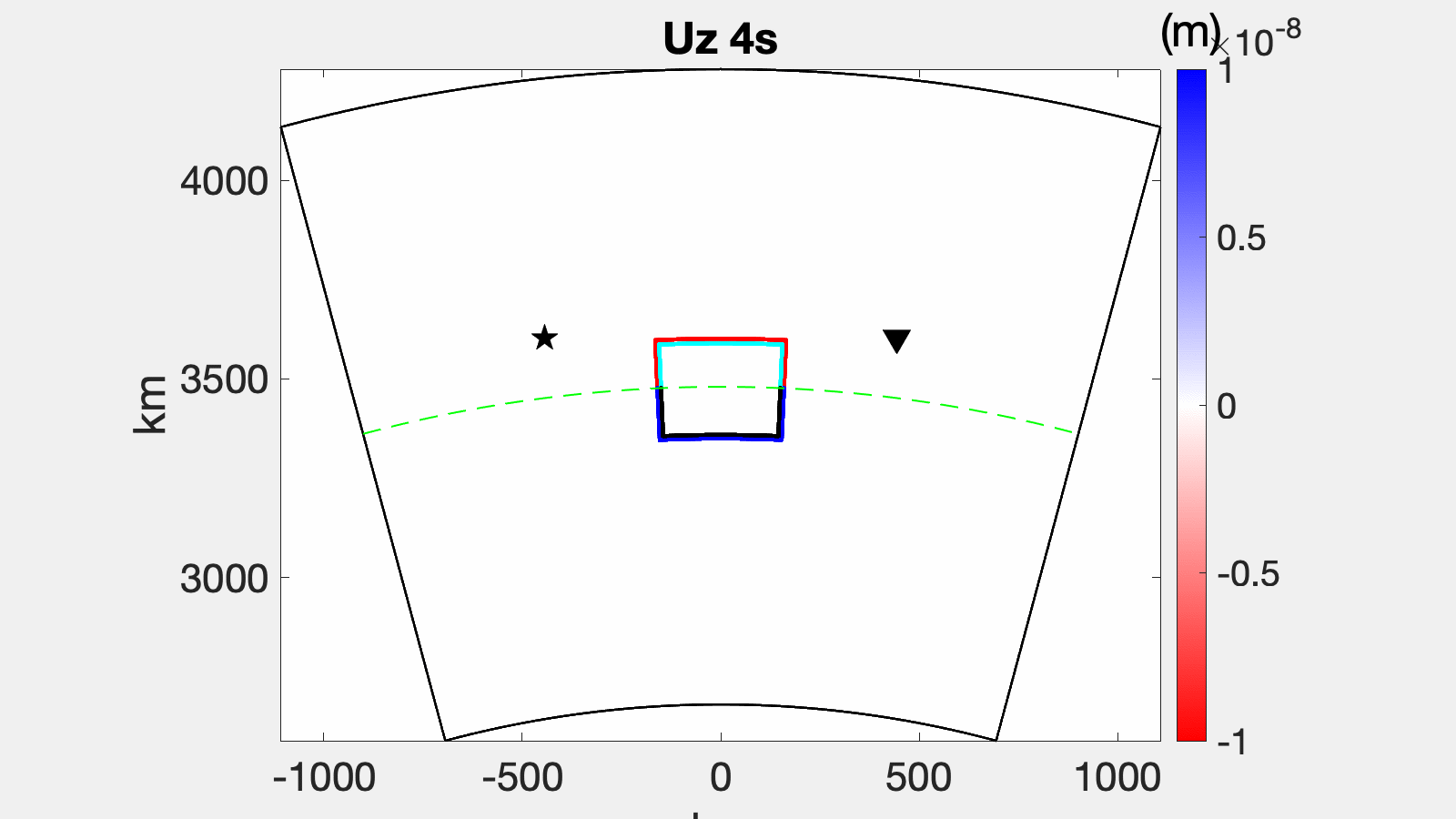

Supplement: Supplementary file 6 — Supplementary Movie 4 [file 41467_2025_56530_MOESM6_ESM.gif]

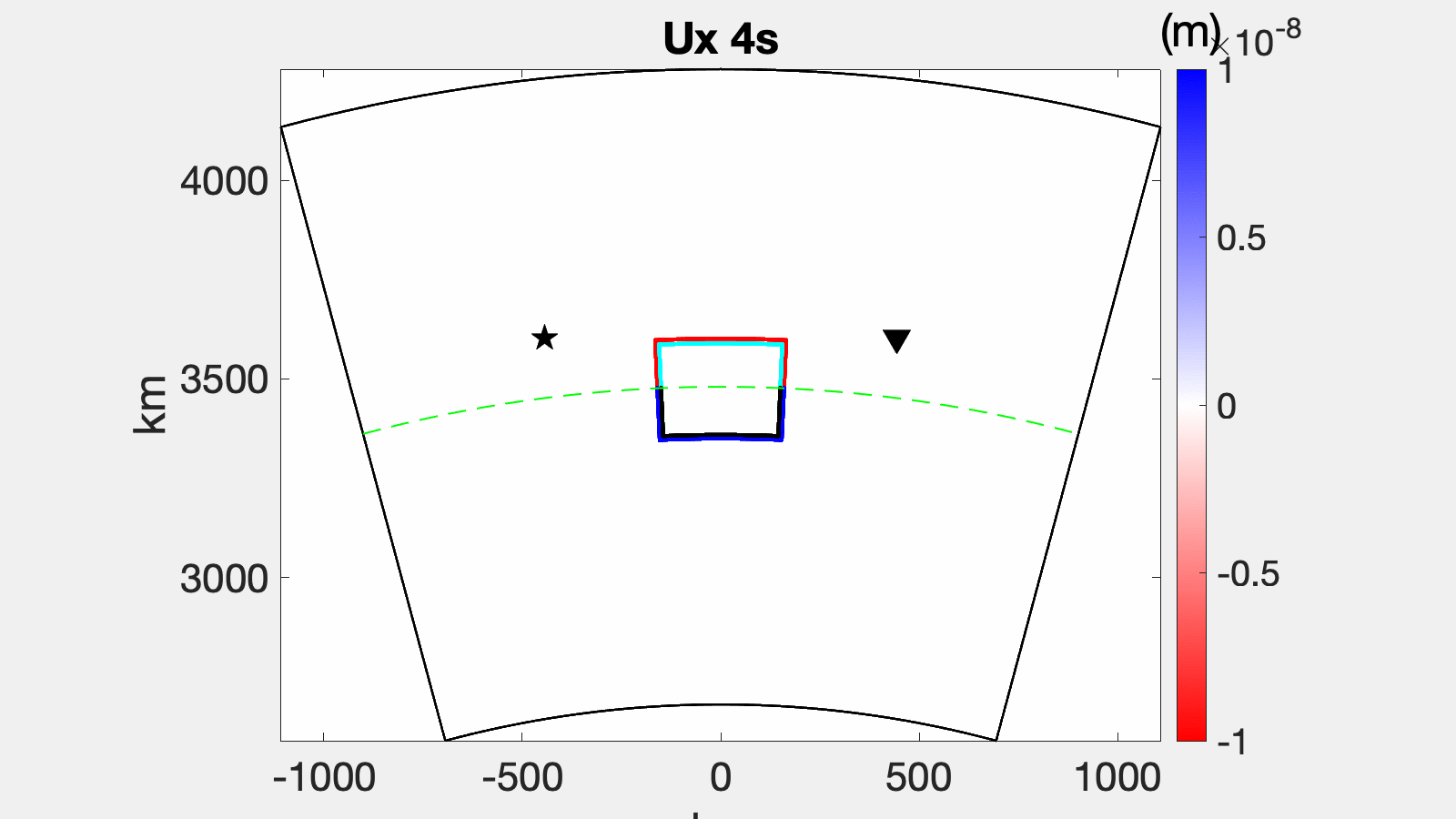

Supplement: Supplementary file 7 — Supplementary Movie 5 [file 41467_2025_56530_MOESM7_ESM.gif]

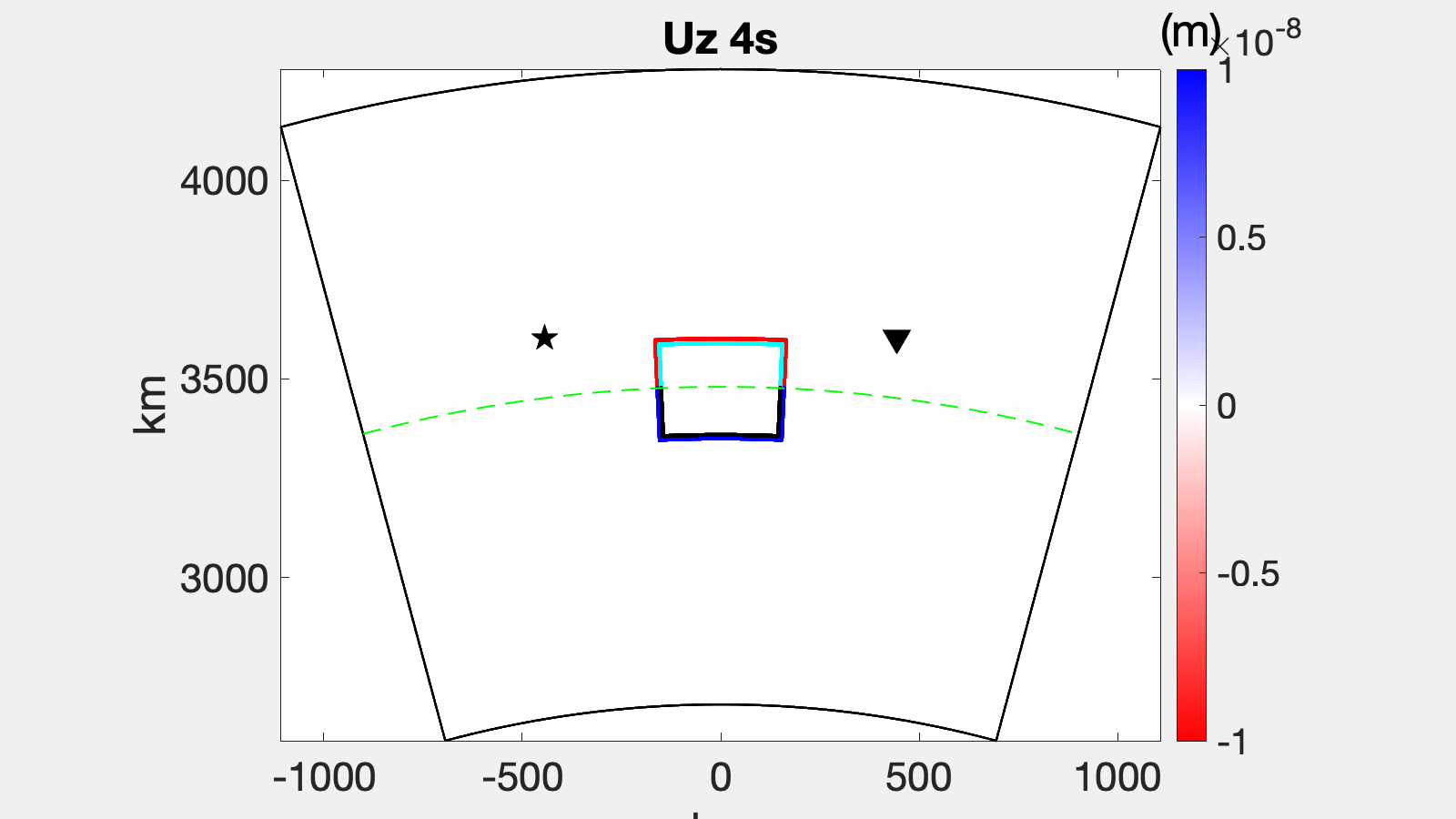

Supplement: Supplementary file 8 — Supplementary Movie 6 [file 41467_2025_56530_MOESM8_ESM.gif]

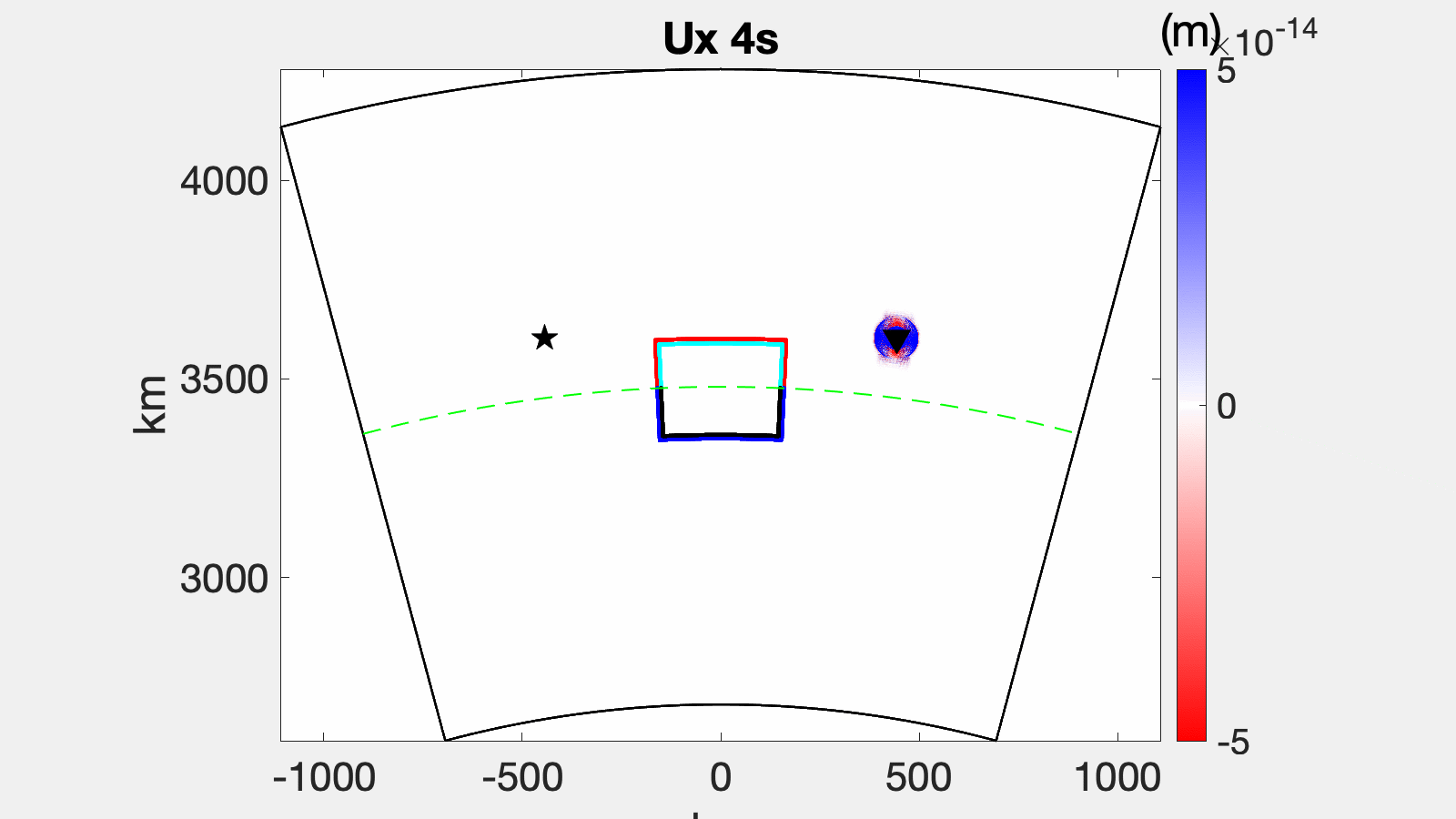

Supplement: Supplementary file 9 — Supplementary Movie 7 [file 41467_2025_56530_MOESM9_ESM.gif]

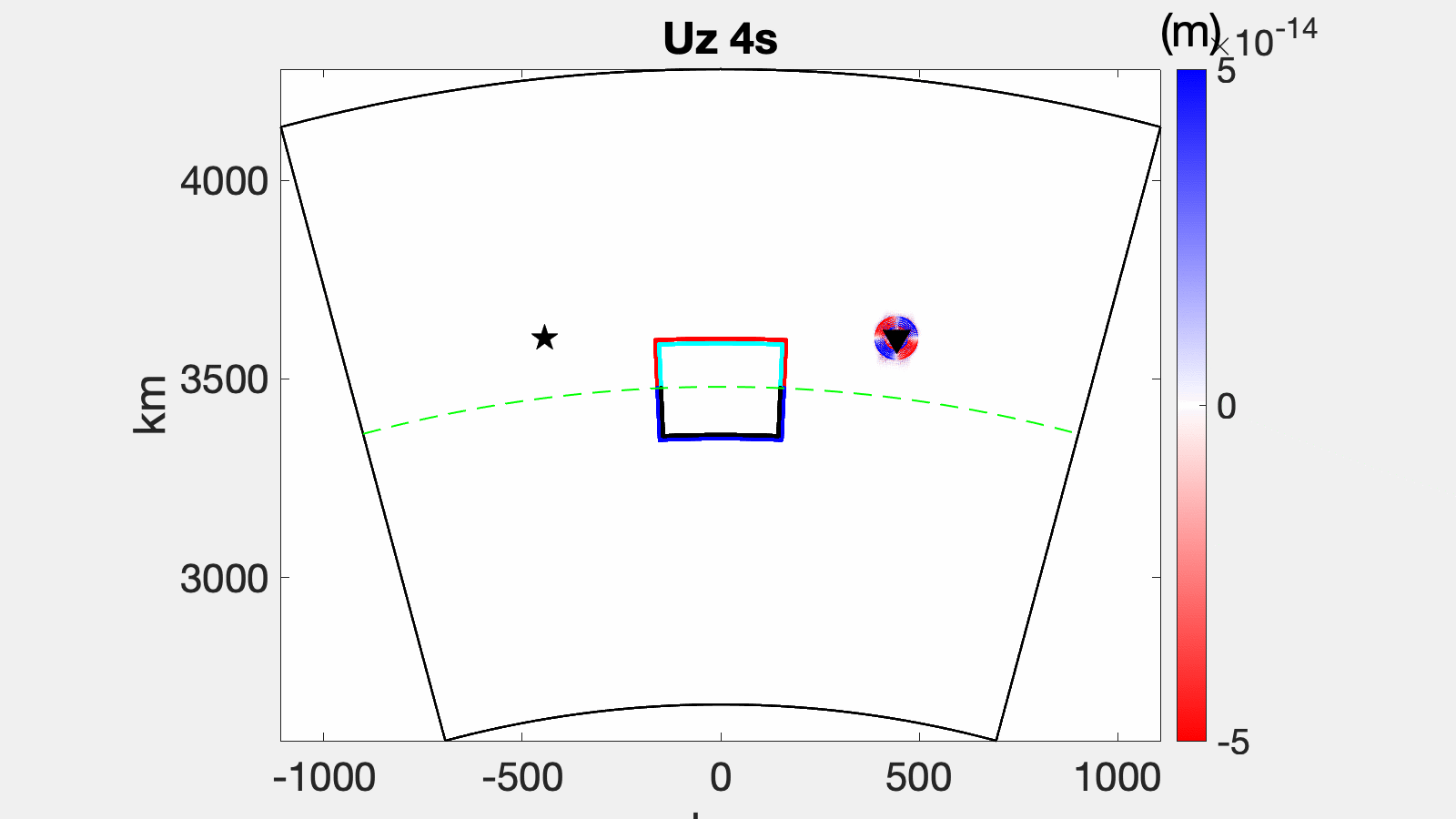

Supplement: Supplementary file 10 — Supplementary Movie 8 [file 41467_2025_56530_MOESM10_ESM.gif]

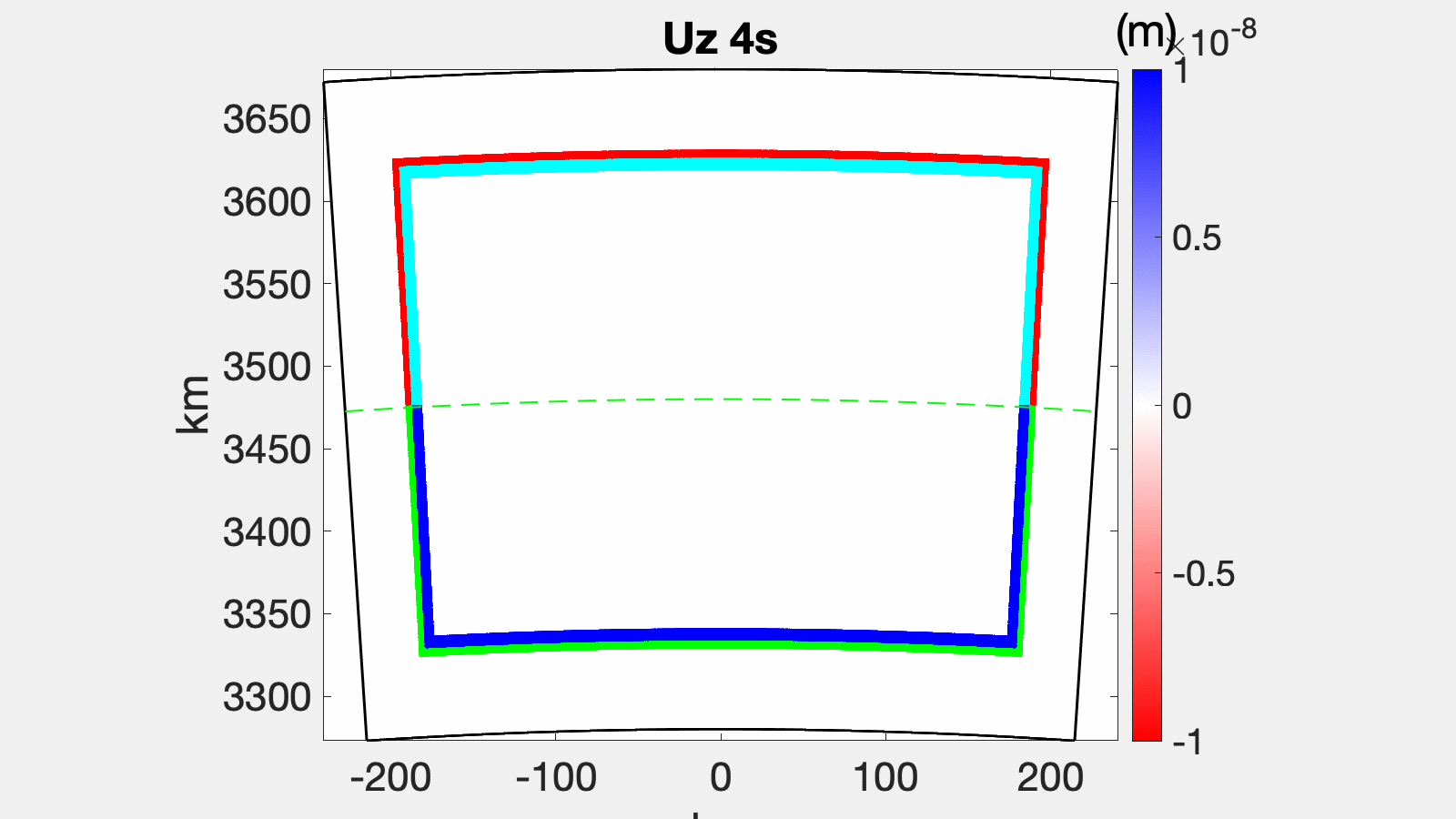

Supplement: Supplementary file 11 — Supplementary Movie 9 [file 41467_2025_56530_MOESM11_ESM.gif]

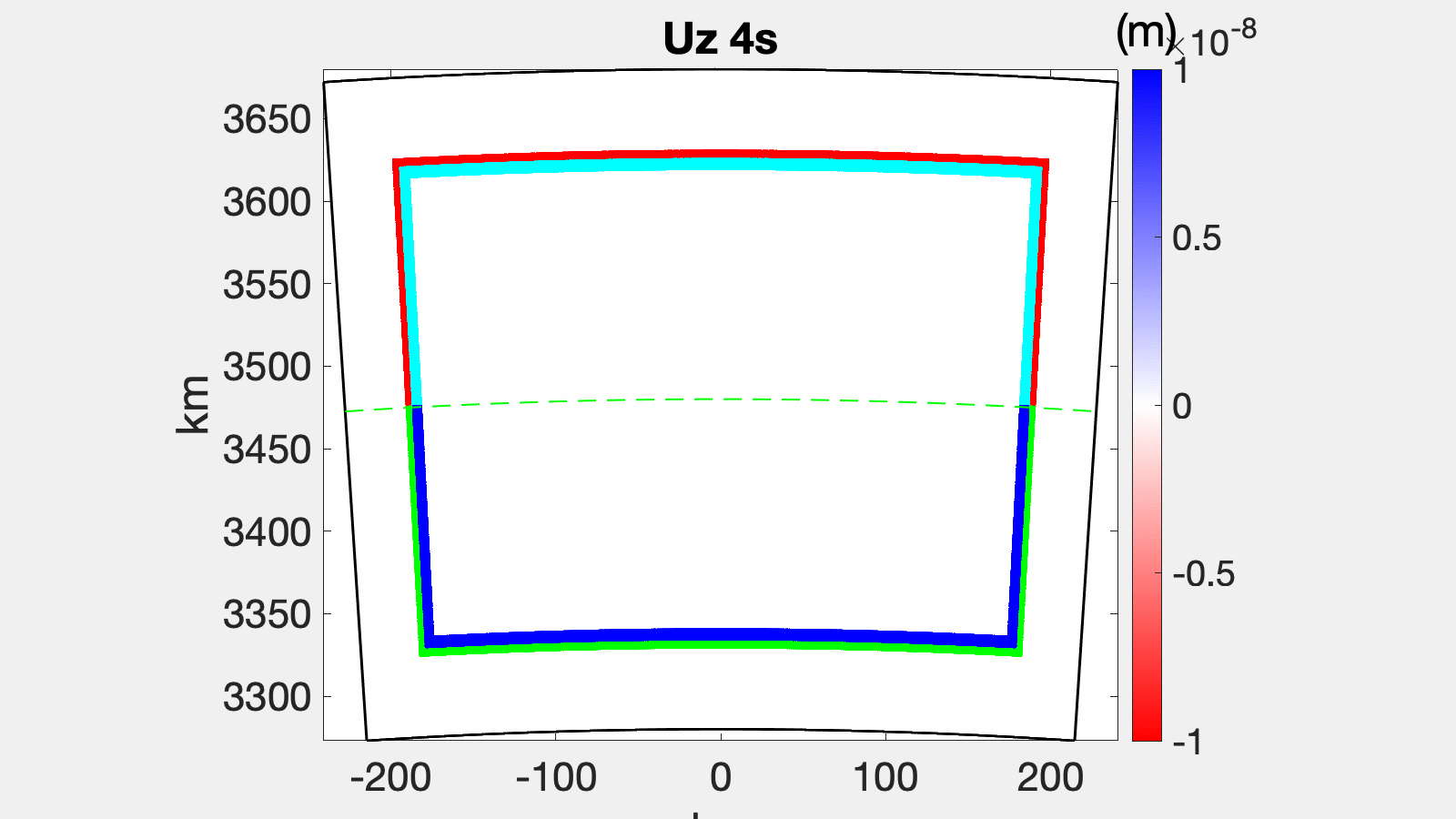

Supplement: Supplementary file 12 — Supplementary Movie 10 [file 41467_2025_56530_MOESM12_ESM.gif]

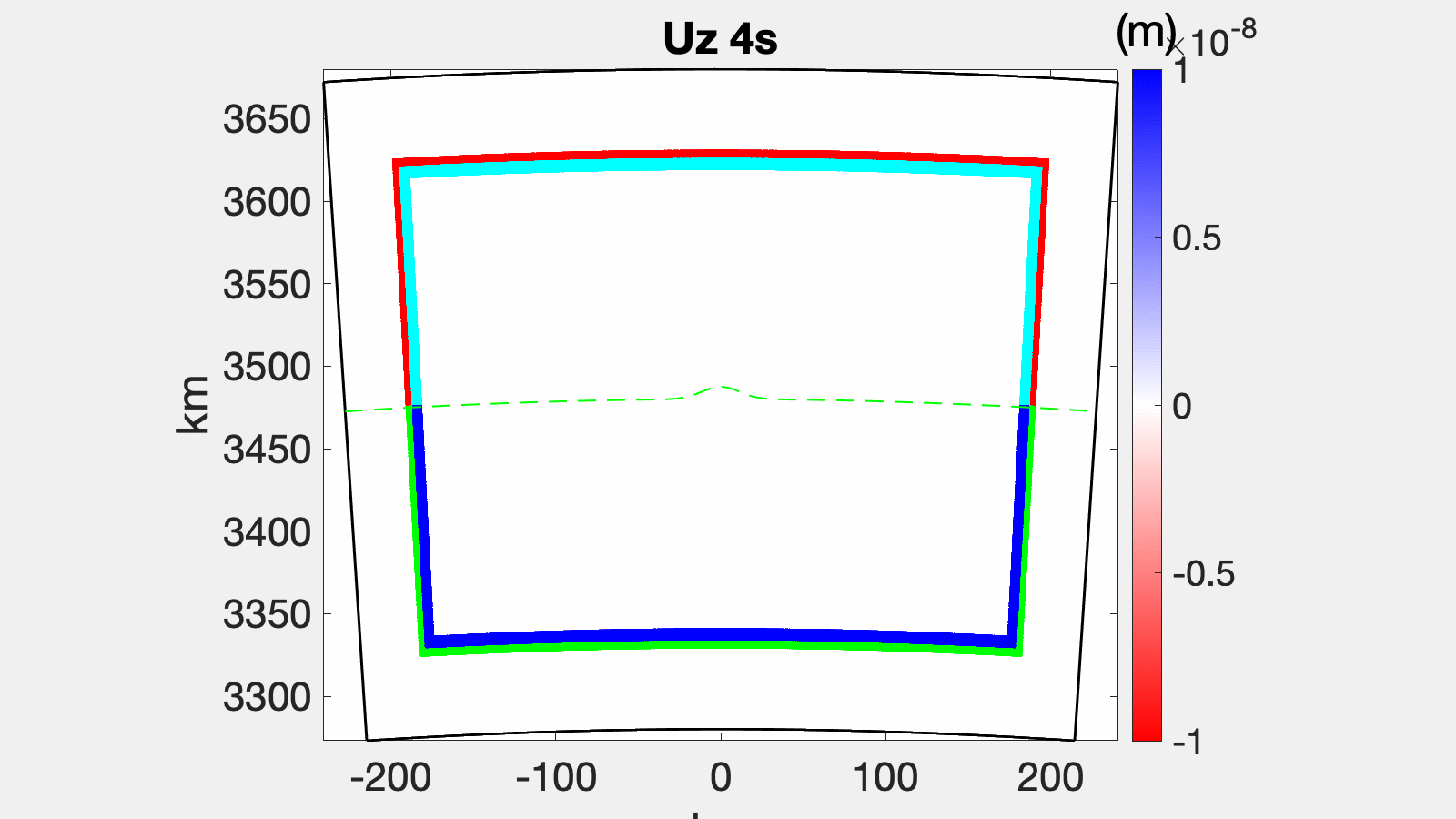

Supplement: Supplementary file 13 — Supplementary Movie 11 [file 41467_2025_56530_MOESM13_ESM.gif]

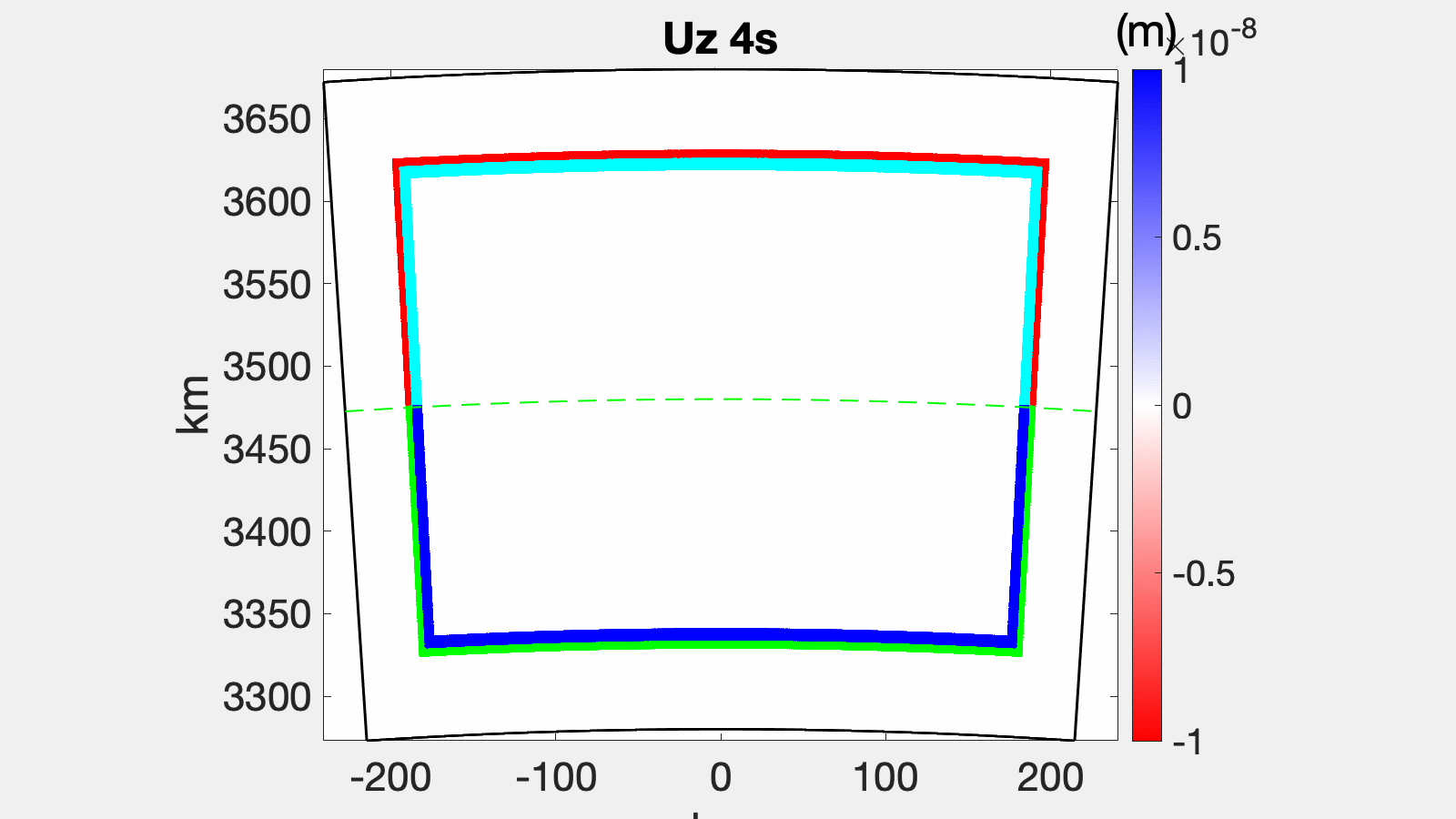

Supplement: Supplementary file 14 — Supplementary Movie 12 [file 41467_2025_56530_MOESM14_ESM.gif]

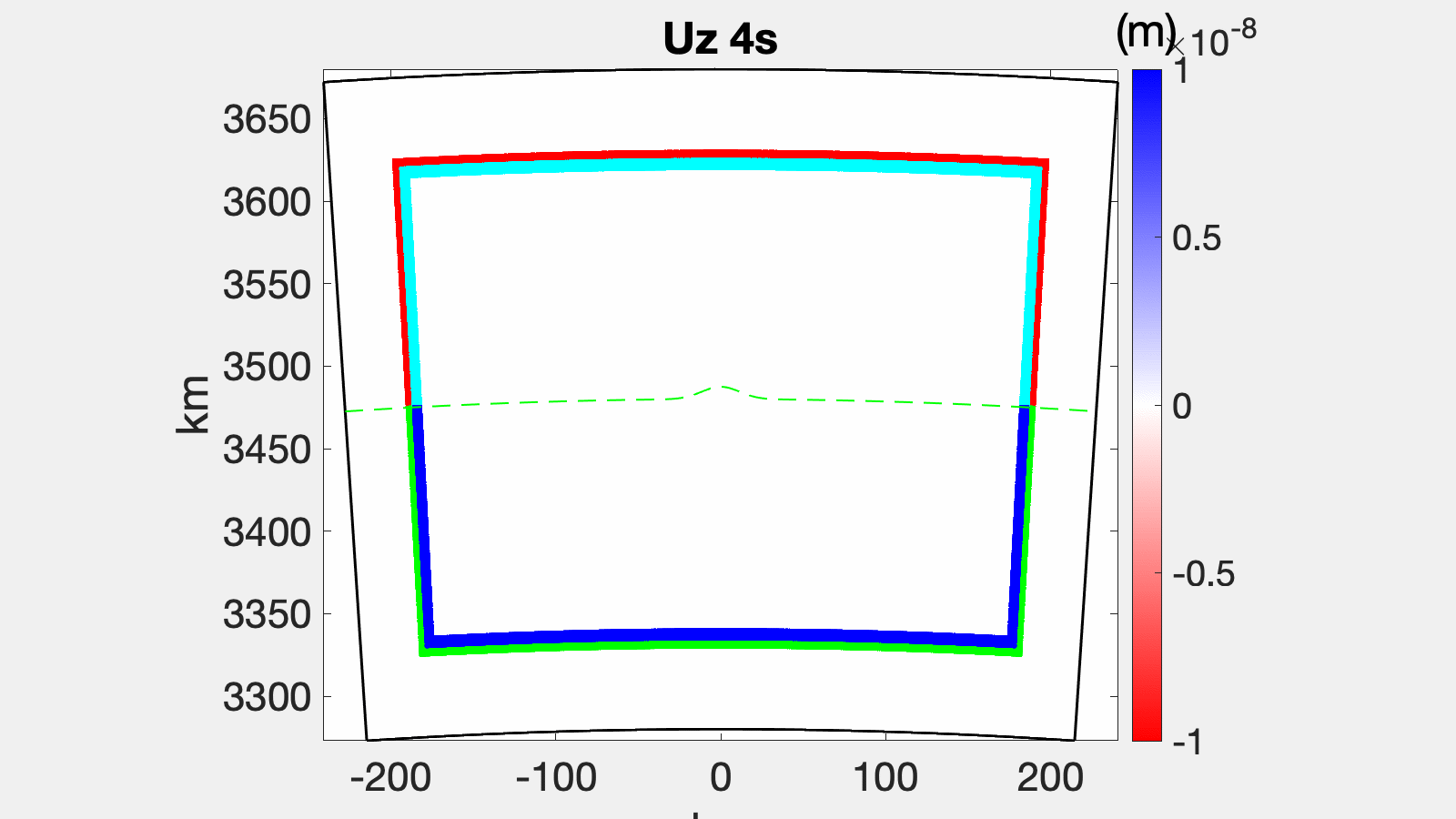

Supplement: Supplementary file 15 — Supplementary Movie 13 [file 41467_2025_56530_MOESM15_ESM.gif]

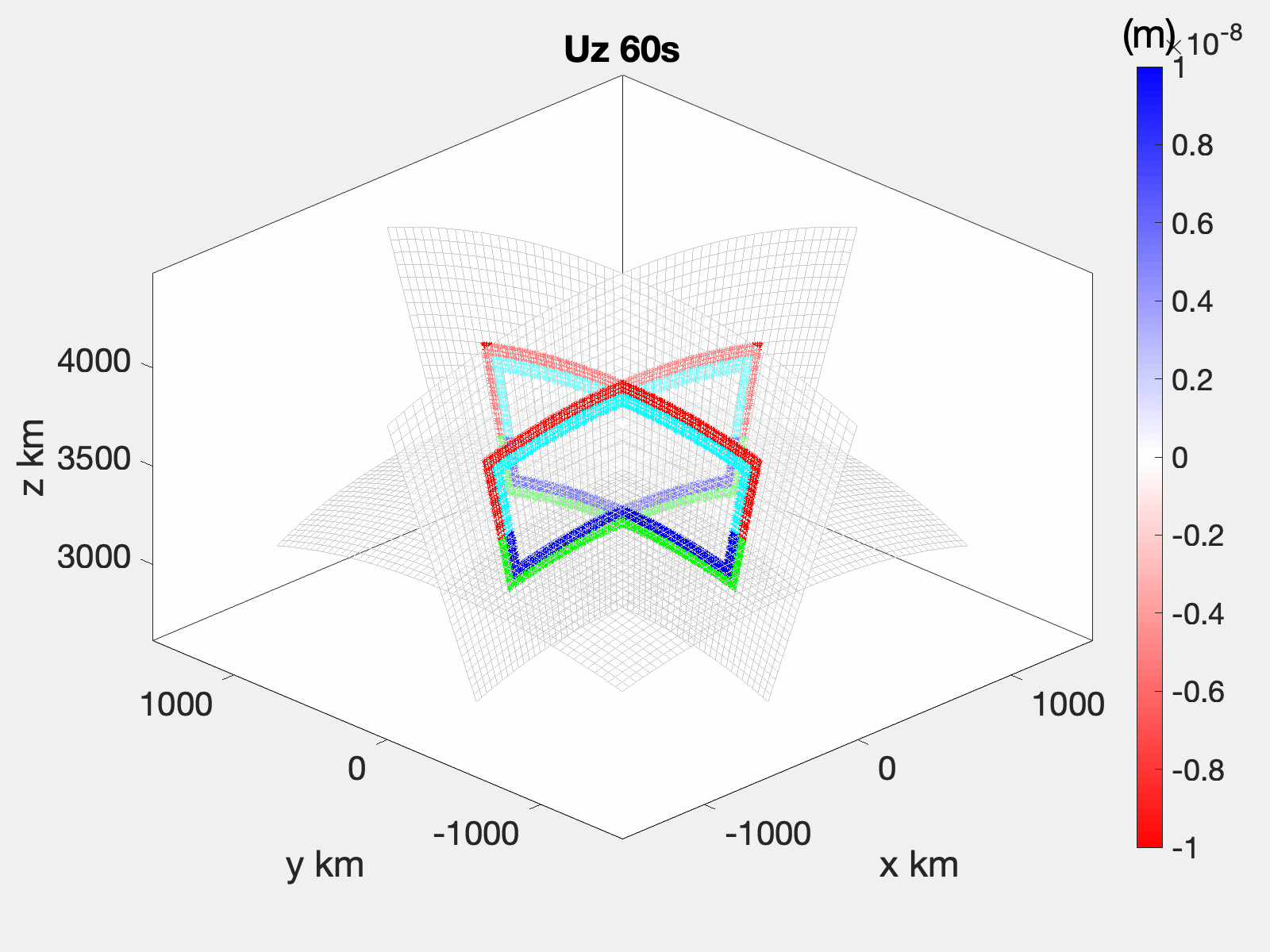

Supplement: Supplementary file 16 — Supplementary Movie 14 [file 41467_2025_56530_MOESM16_ESM.gif]

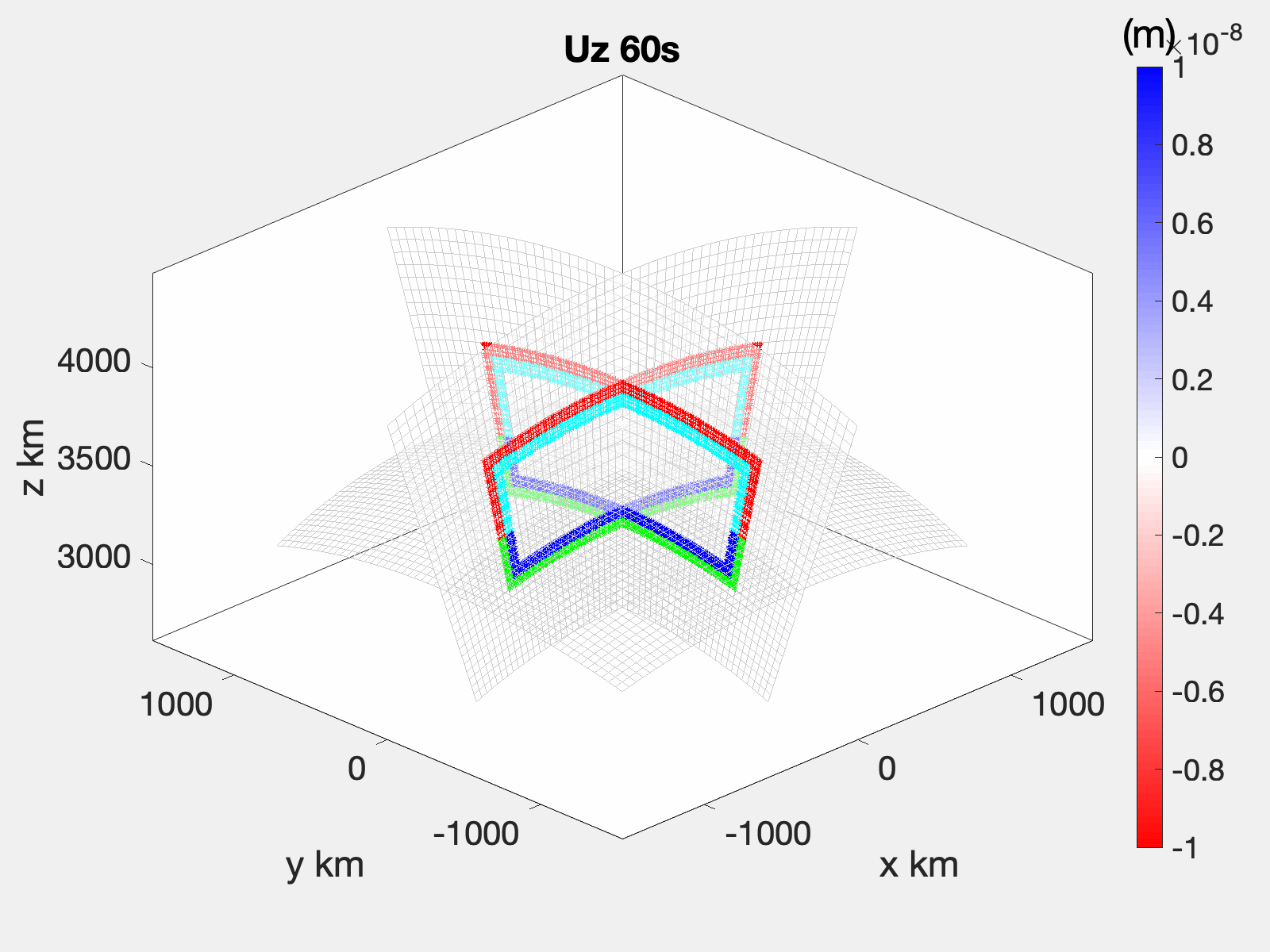

Supplement: Supplementary file 17 — Supplementary Movie 15 [file 41467_2025_56530_MOESM17_ESM.gif]

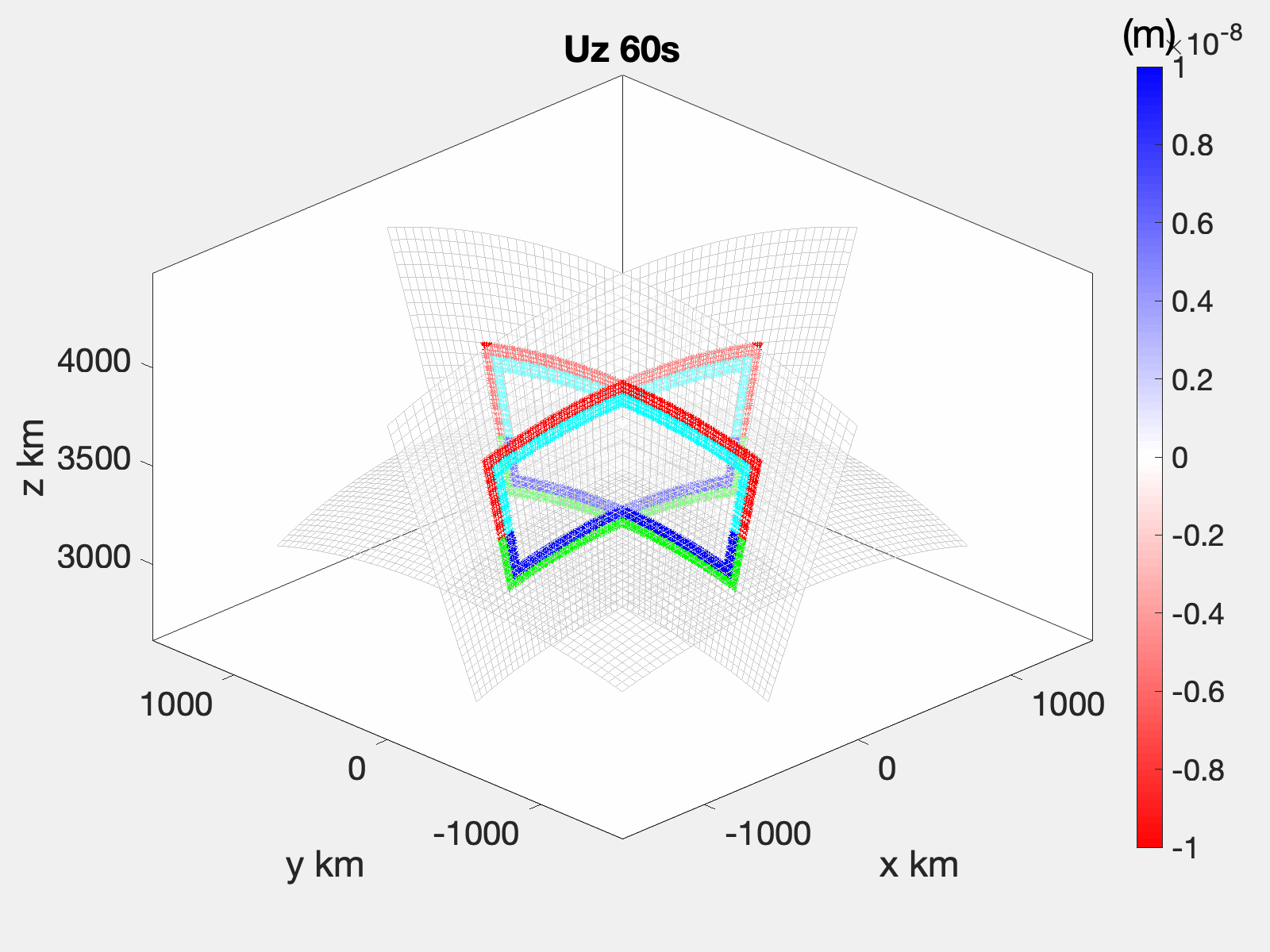

Supplement: Supplementary file 18 — Supplementary Movie 16 [file 41467_2025_56530_MOESM18_ESM.gif]

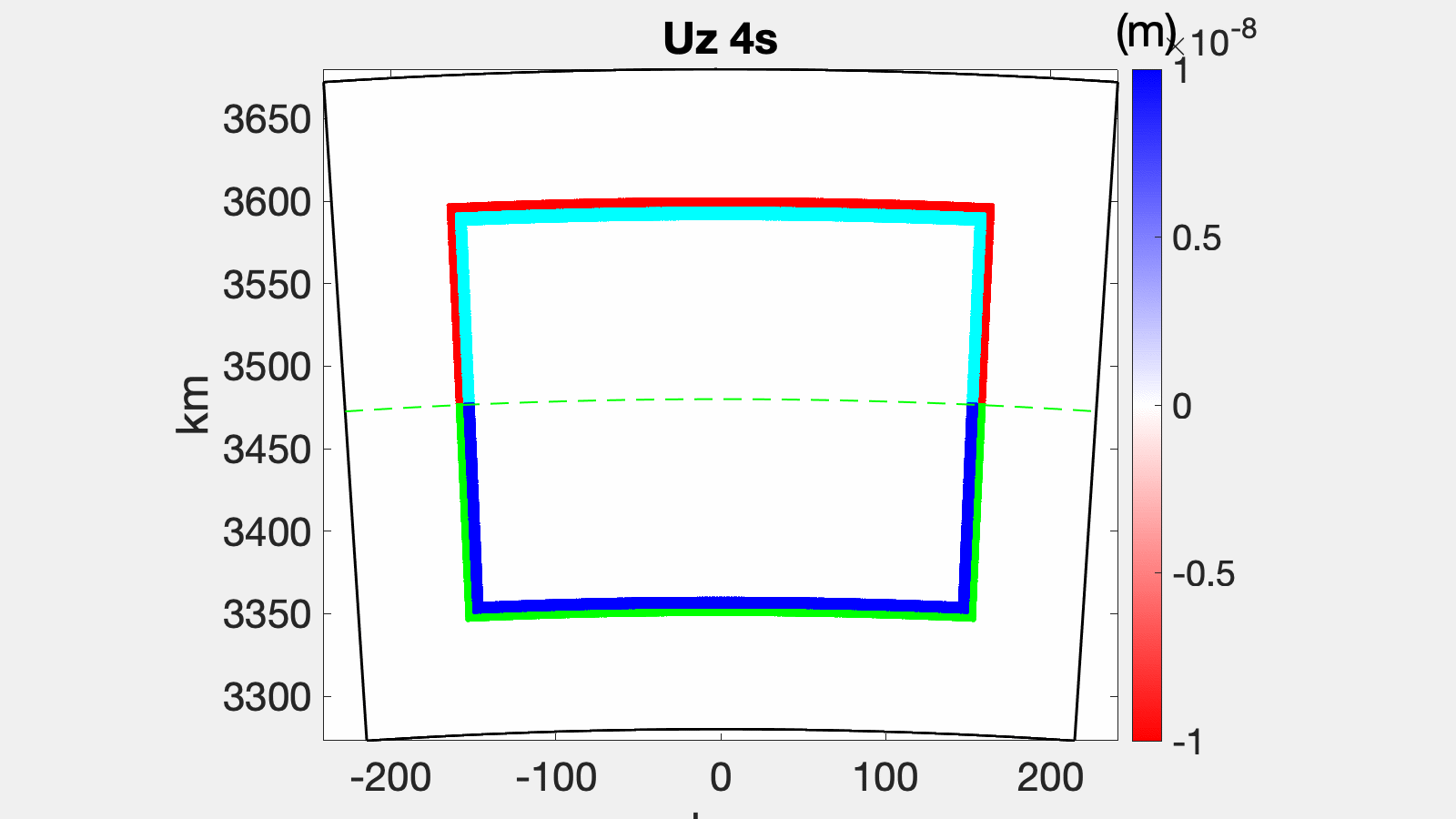

Supplement: Supplementary file 19 — Supplementary Movie 17 [file 41467_2025_56530_MOESM19_ESM.gif]

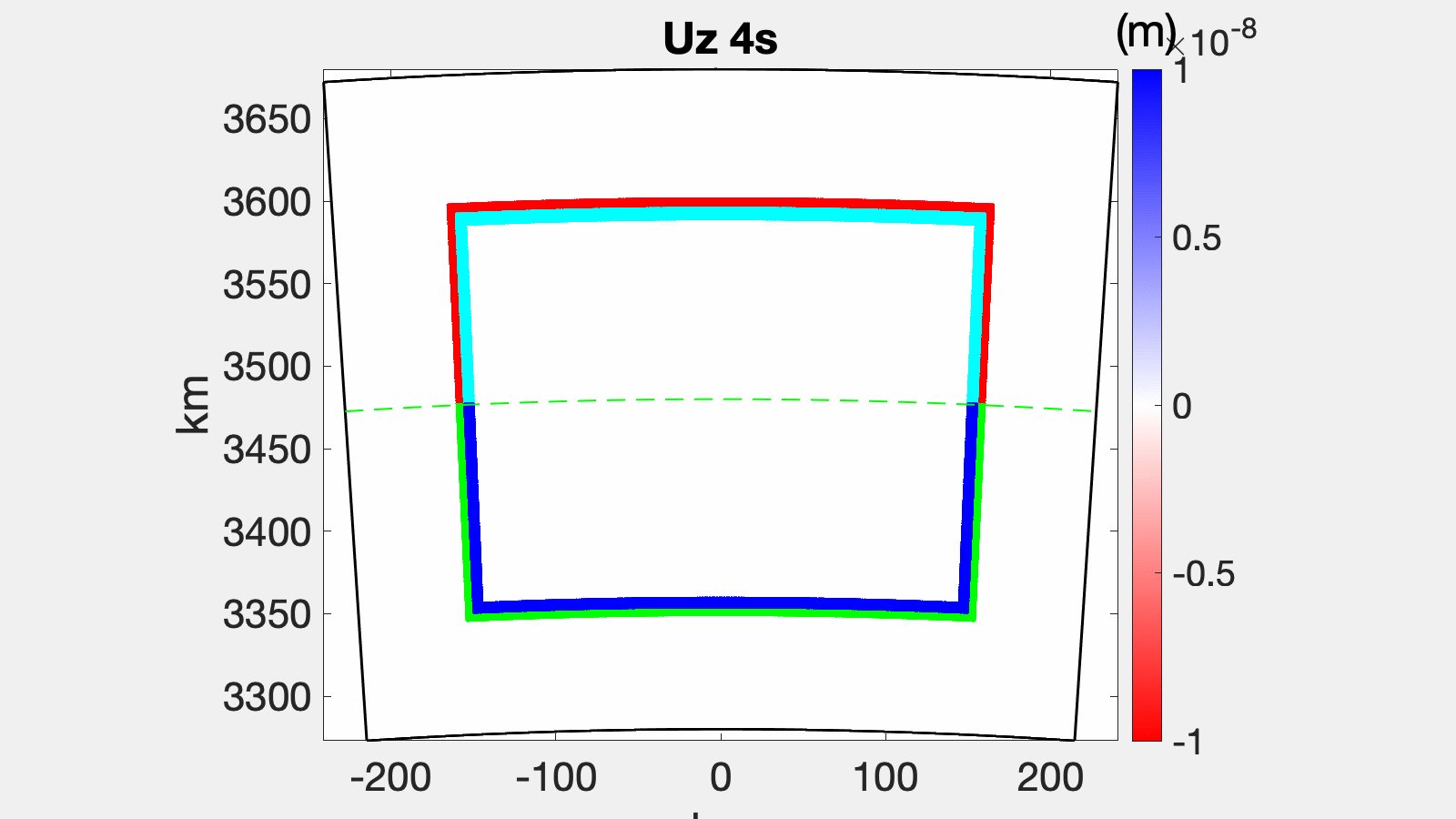

Supplement: Supplementary file 20 — Supplementary Movie 18 [file 41467_2025_56530_MOESM20_ESM.gif]

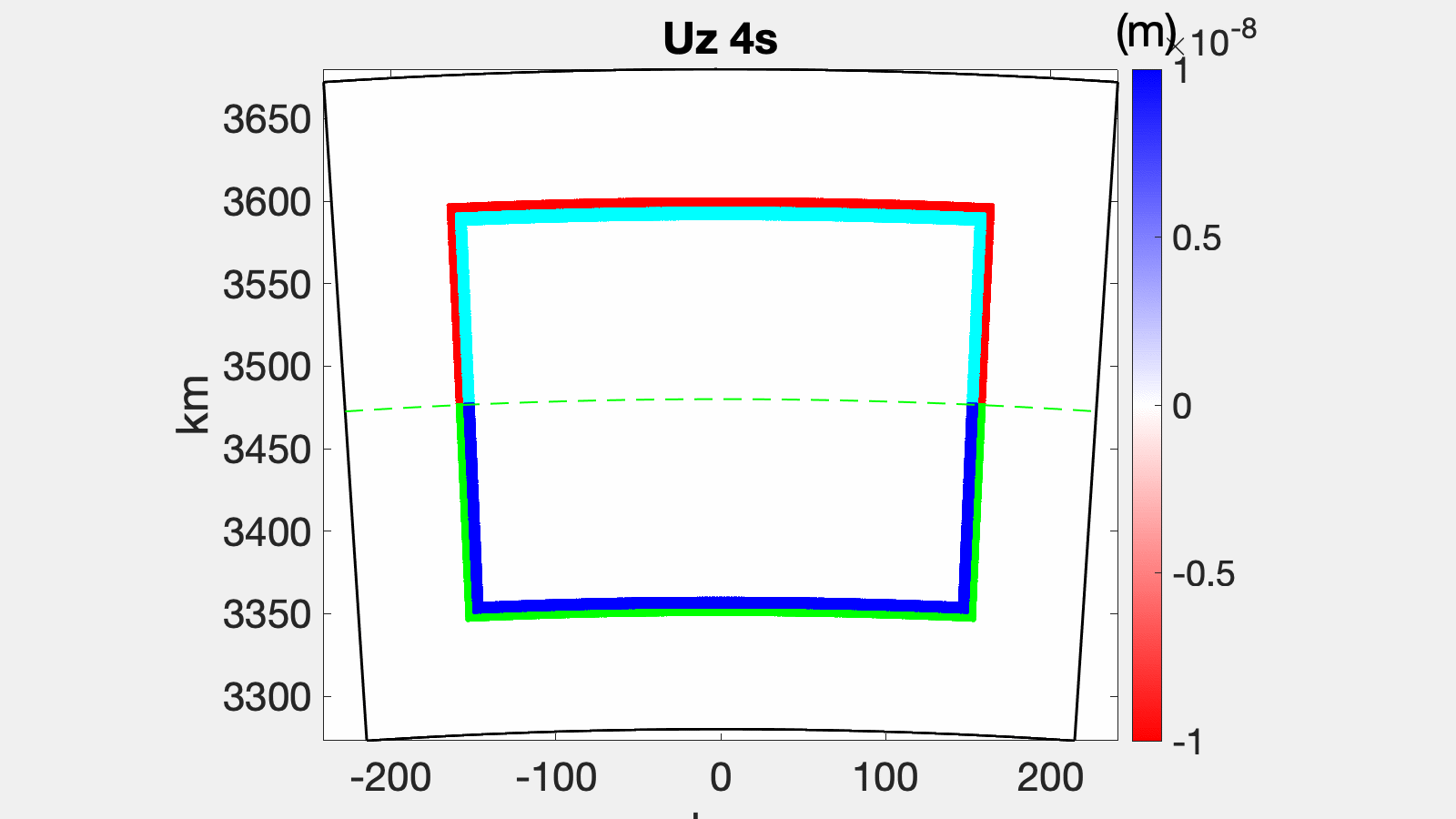

Supplement: Supplementary file 21 — Supplementary Movie 19 [file 41467_2025_56530_MOESM21_ESM.gif]

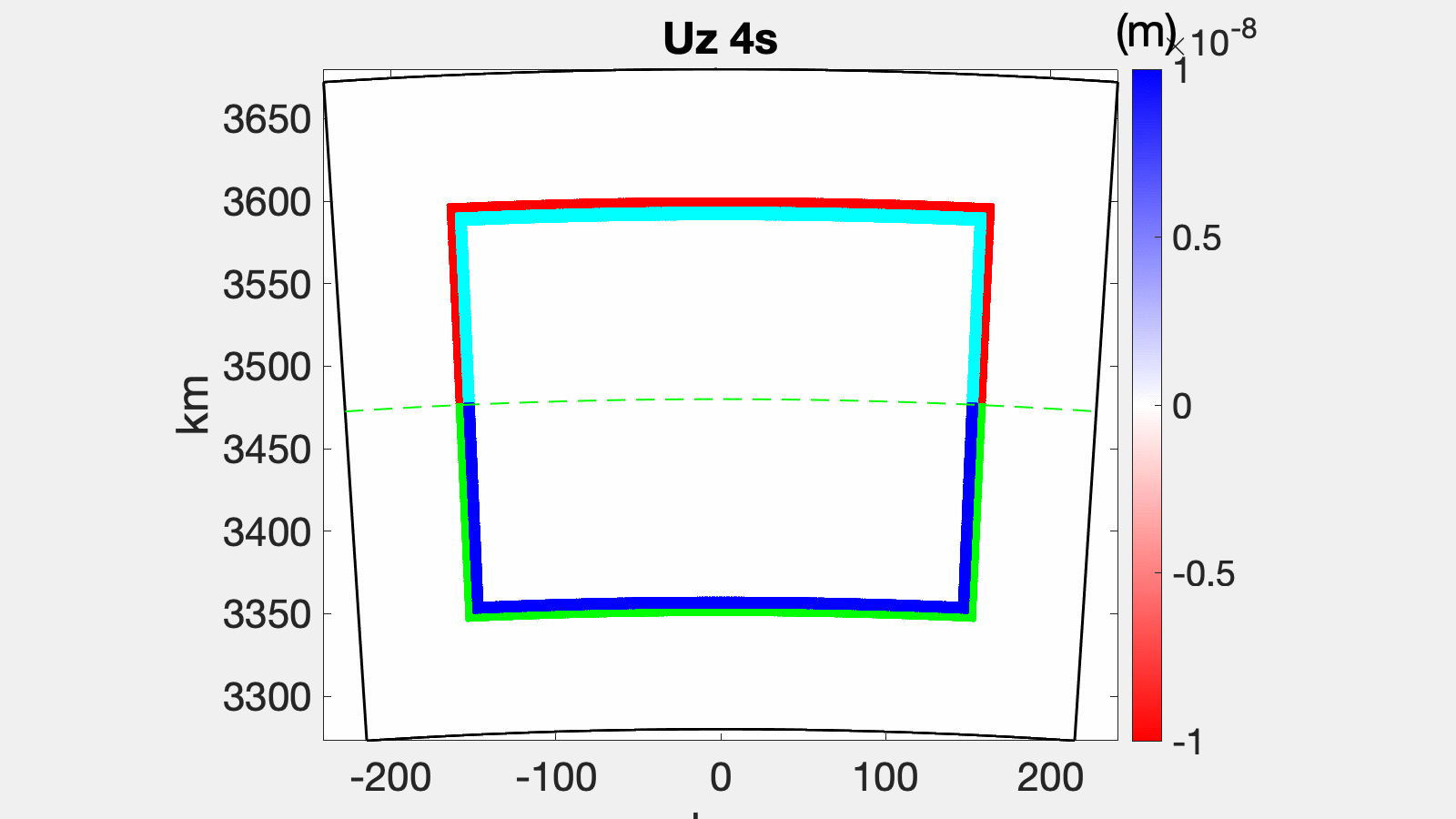

Supplement: Supplementary file 22 — Supplementary Movie 20 [file 41467_2025_56530_MOESM22_ESM.gif]

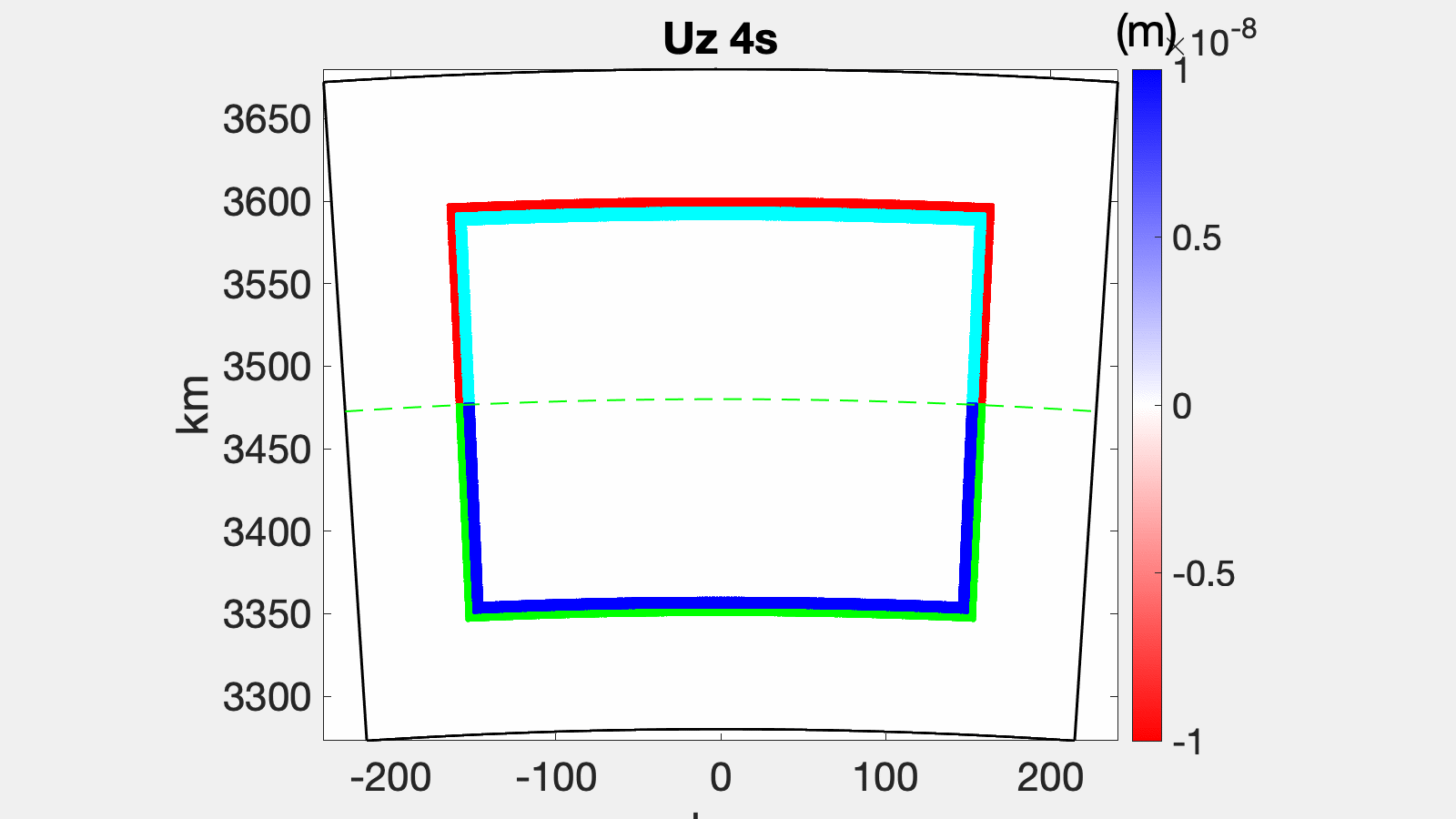

Supplement: Supplementary file 23 — Supplementary Movie 21 [file 41467_2025_56530_MOESM23_ESM.gif]

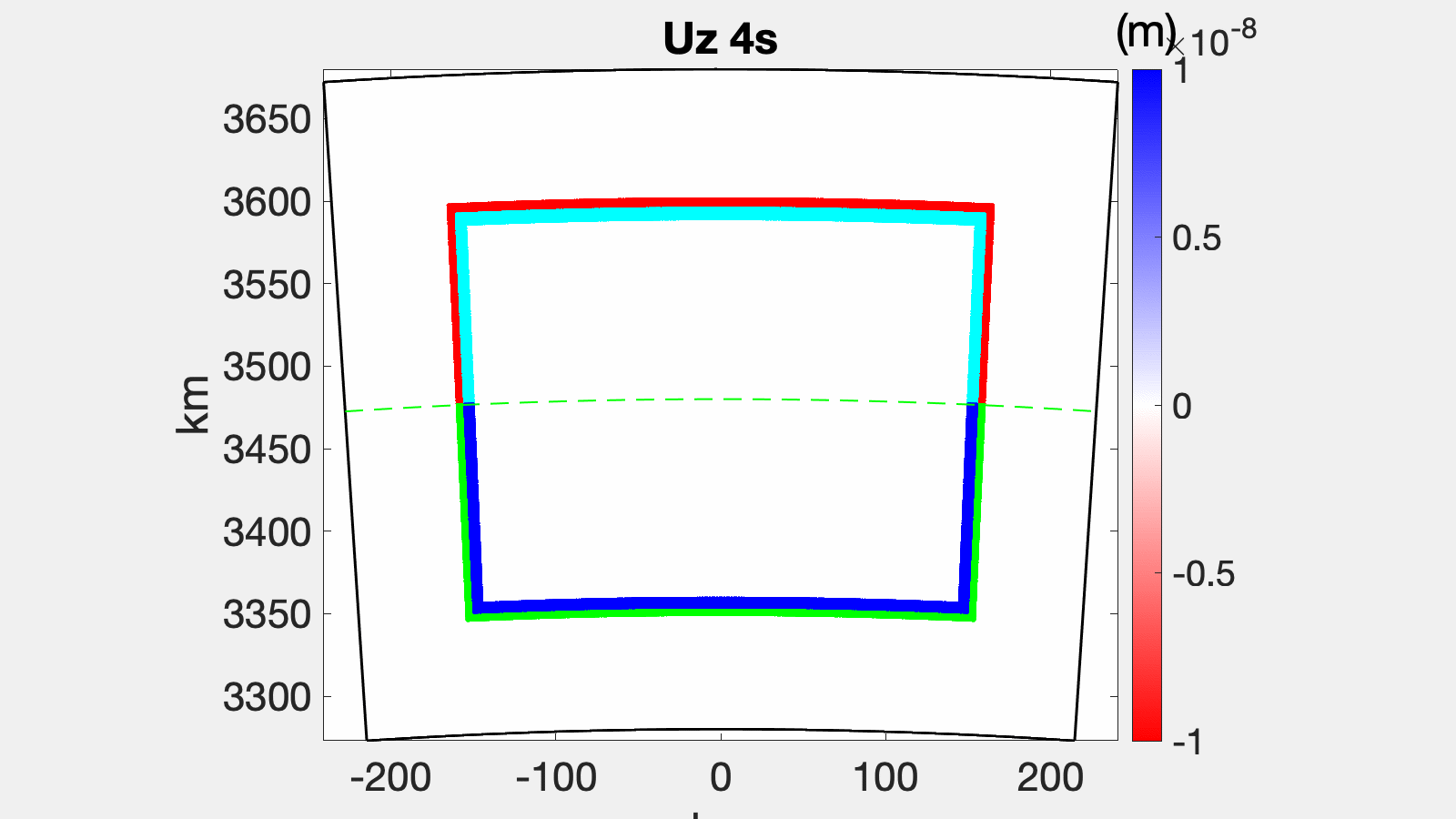

Supplement: Supplementary file 24 — Supplementary Movie 22 [file 41467_2025_56530_MOESM24_ESM.gif]

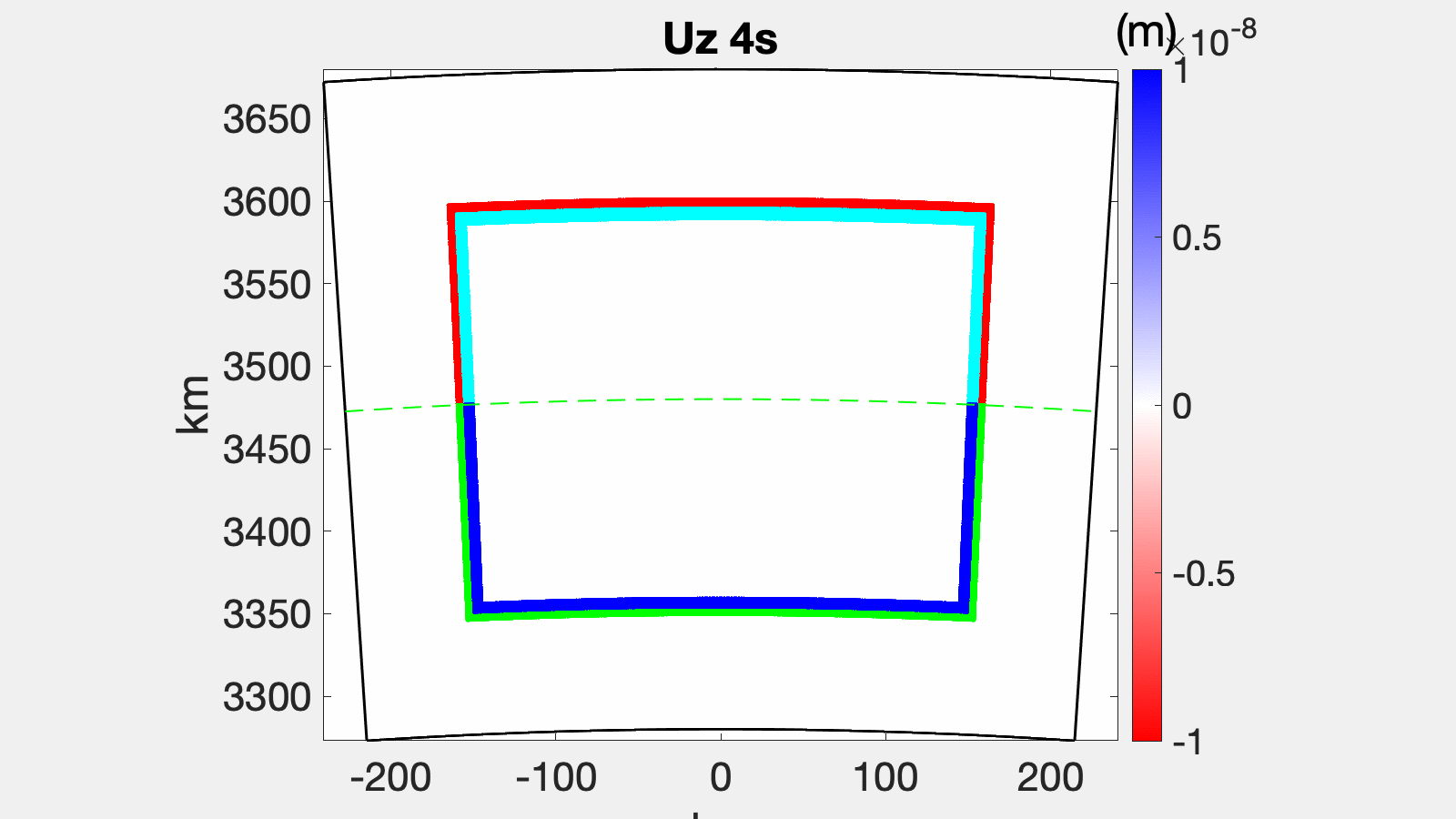

Supplement: Supplementary file 25 — Supplementary Movie 23 [file 41467_2025_56530_MOESM25_ESM.gif]
